# Imprinted Hydrogel Nanoparticles for Protein Biosensing: A Review

**DOI:** 10.1021/acssensors.3c01010

**Published:** 2023-08-09

**Authors:** Ana T. Silva, Rui Figueiredo, Manuel Azenha, Pedro A.S. Jorge, Carlos M. Pereira, José A. Ribeiro

**Affiliations:** † CIQUP/IMS, Department of Chemistry and Biochemistry, Faculty of Sciences, University of Porto, Rua do Campo Alegre 687, s/n, Porto 4169-007, Portugal; ‡ INESC TEC−Institute for Systems and Computer Engineering, Technology and Science, Faculty of Sciences, University of Porto, 4169-007 Porto, Portugal; § Department of Physics and Astronomy, Faculty of Sciences, University of Porto, Rua do Campo Alegre 687, s/n, Porto 4169-007, Portugal

**Keywords:** hydrogels, biomimetic, molecularly imprinted nanoparticles (MIP NPs), nanoMIPs, nanogels, biosensing, proteins, epitope imprinting, precipitation polymerization, solid-phase synthesis

## Abstract

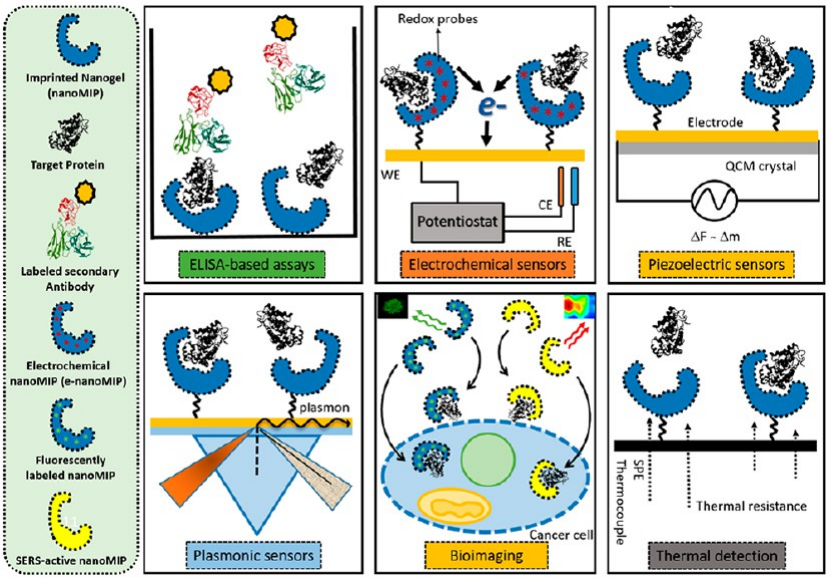

Over the past decade, molecular imprinting (MI) technology has made tremendous progress, and the advancements in nanotechnology have been the major driving force behind the improvement of MI technology. The preparation of nanoscale imprinted materials, i.e., molecularly imprinted polymer nanoparticles (MIP NPs, also commonly called nanoMIPs), opened new horizons in terms of practical applications, including in the field of sensors. Currently, hydrogels are very promising for applications in bioanalytical assays and sensors due to their high biocompatibility and possibility to tune chemical composition, size (microgels, nanogels, etc.), and format (nanostructures, MIP film, fibers, etc.) to prepare optimized analyte-responsive imprinted materials. This review aims to highlight the recent progress on the use of hydrogel MIP NPs for biosensing purposes over the past decade, mainly focusing on their incorporation on sensing devices for detection of a fundamental class of biomolecules, the peptides and proteins. The review begins by directing its focus on the ability of MIPs to replace biological antibodies in (bio)analytical assays and highlight their great potential to face the current demands of chemical sensing in several fields, such as disease diagnosis, food safety, environmental monitoring, among others. After that, we address the general advantages of nanosized MIPs over macro/micro-MIP materials, such as higher affinity toward target analytes and improved binding kinetics. Then, we provide a general overview on hydrogel properties and their great advantages for applications in the field of Sensors, followed by a brief description on current popular routes for synthesis of imprinted hydrogel nanospheres targeting large biomolecules, namely precipitation polymerization and solid-phase synthesis, along with fruitful combination with epitope imprinting as reliable approaches for developing optimized protein-imprinted materials. In the second part of the review, we have provided the state of the art on the application of MIP nanogels for screening macromolecules with sensors having different transduction modes (optical, electrochemical, thermal, etc.) and design formats for single use, reusable, continuous monitoring, and even multiple analyte detection in specialized laboratories or *in situ* using mobile technology. Finally, we explore aspects about the development of this technology and its applications and discuss areas of future growth.

MI technology is a process for creating synthetic receptors that exhibit high affinity and selectivity toward specific molecules or other structures. The imprinting process involves the formation of a polymer matrix around a template molecule, which is subsequently removed, leaving behind a cavity that is complementary in size, shape, and functional groups to the template molecule (see Figure 1).

**Figure 1:**
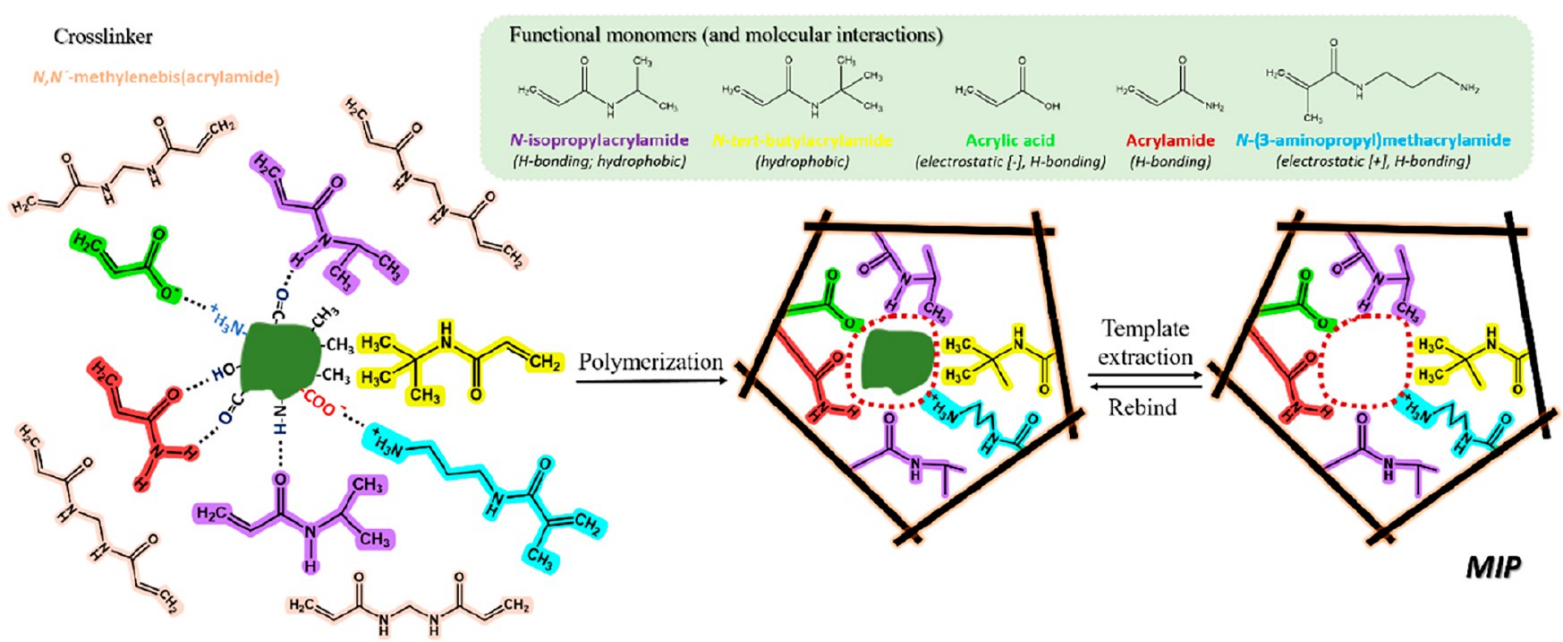
Schematic representation of the MI process. First, the functional monomers interact with the template molecule in solution to create a specific cavity. Then, cross-linker and initiator are added for polymerization. Finally, the extraction of template molecule from the polymeric matrix gives origin to empty imprinted cavities with the ability to specifically rebind the target analyte.

MI technology has been developed since the 1930s, when Pauling and his colleagues first proposed the concept of molecular recognition based on complementary shape and charge distribution of molecules.^1^ In 1972, Wulff demonstrated the synthesis of a polymer with recognition properties for amino acids and peptides.^1^ In the early 1980s, a new approach to molecular imprinting was introduced by Mosbach and collaborators, known as noncovalent imprinting.^2^ In these early stages of MI, the development of synthetic receptors was limited by the lack of suitable polymerization techniques. However, the field of MI began to rapidly advance with the introduction of new polymerization methods in the 1980s and 1990s, such as the free radical polymerization and the reversible addition–fragmentation chain transfer (RAFT) polymerization.

Radical polymerization is the most commonly used approach for the synthesis of MIPs. The resulting MIPs are highly cross-linked and exhibit excellent stability and mechanical properties.^3^ Sol–gel synthesis is another popular approach for the synthesis of MIPs^4^ involving the hydrolysis and condensation of metal alkoxides to form a sol which is subsequently gelled to form a 3D network. The prepared MIPs have high surface area and porosity, making them suitable for chromatography applications, among others. Meanwhile, electrochemical polymerization^5−7^ emerged in the literature as a “smart approach” for the synthesis of MIPs by simply electropolymerizing redox functional monomers in the presence of a template molecule, resulting in MIP thin films with excellent surface properties that can be easily integrated into electronic devices. Over recent years, bioimprinting became a promising sustainable technique for the synthesis of MIPs using biomolecules (proteins, polysaccharides, etc.) as building blocks, having the potential to overcome the limitations of conventional formats, such as poor biocompatibility and solubility.^8^

Overall, MI technology has made remarkable progress since its inception, greatly expanding the spectrum of applications for MIPs. The scope of this review is to discuss recent advancements in the production of hydrogel MIP NPs for biosensing applications, with a particular emphasis on their integration into sensing devices for detecting peptides and proteins.

## MIPs As an Alternative to Biological Antibodies

Biological antibodies have been extensively used in life sciences and biosensing assays. Although they provide very specific interaction through the formation of stable complexes with the target antigen, these bioreceptors commonly suffer from lack of long-term stability, can irreversibly denaturate, require careful handling/storage conditions, the acquisition costs are very high and have associated ethical dilemmas. Due to these unfavorable properties, MIPs are currently a very promising alternative to their biological counterparts.^9,10^ In fact, there are several advantages of using MIPs (“plastic antibodies”) in bioassays for sensing applications (see Table 1). They are synthetic biomimetic materials that can selectively bind to the analytes of interest, offering long-term stability and low production cost in short time.^9,10^

**Table 1:** Comparison of Properties between Biological Antibodies and MIPs

characteristic	antibodies	MIPs
production time	few months	few days to weeks
thermal stability	denaturate at ≈70 °C	resistant up to 140 °C
pH stability	low	high
stability	6–12 months	years
production method	animal immunization	chemical synthesis
amount of target molecules	medium	high
storage	freezer	from 6 °C to RT
price	high	low
selectivity/specificity/affinity	high (even in complex matrices; *K*_D_ often <1 nM for monoclonal antibodies)	low to high (IF from nearly 1 to values >10; *K*_D_ in the nM to pM range for nanosized MIPs)

The synergetic combination between the fields of MI and Sensors Development is already well established. The general use of MIPs (bulk, microparticles, films, etc.) as synthetic receptors in sensing platforms for (bio)analysis already found relevant practical applications in disease diagnosis,^11^ environmental monitoring,^12^ food safety,^13^ among others, mainly using electrochemical^5,12^ and optical^14^ approaches.

## From Macro- To Micro- To Nanosized MIP Particles

Pioneer works of Wulff^1,15^ and Mosbach^2,16^ were based on simple bulk imprinting leading to the formation of macroscale polymeric structures for diverse applications. Although these bulk materials can be sieved and ground into smaller micro/nano particles, the performance of the resulting heterogeneous MIP materials is often not suitable for precision and/or *in vivo* applications due to (i) broad distribution of binding sites, leading to different affinities for the target molecule; (ii) many possibilities for nonspecific interactions; (iii) high batch-to-batch variability; and (iv) diffusion limitations for large size biomolecules.

**Figure 2:**
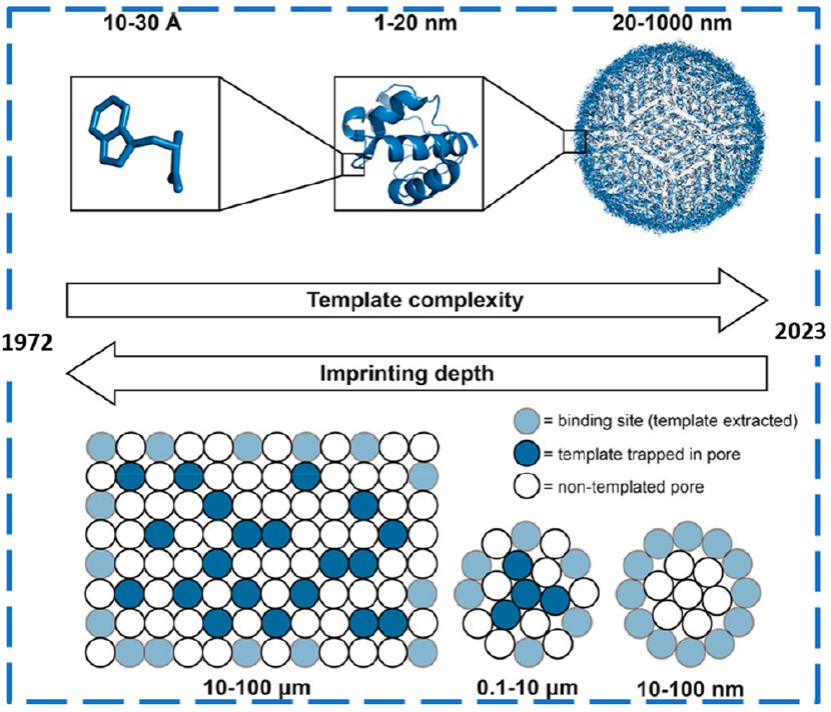
Evolution of MI from bulk materials for recognition of small template molecules to micro- and nanostructured MIPs for large templates, such as proteins and viruses. The term “imprinting depth” refers to the dimensionality of imprinted materials architecture that determines its functions and properties. Adapted from ref (17). Copyright 2017 American Chemical Society.

To overcome some limitations of bulk imprinting, researchers in the MI field started to scale down in MIPs dimension (i.e., the “imprinting depth” of imprinted materials), from macroscale MIPs to microparticles, thin films and nanosized particles, aiming to improve binding sites accessibility, favoring template extraction while increasing the number of imprinted cavities per unit of area. At the same time, molecular imprinting strategies, first applied to small molecular templates, were also successfully developed for imprinting of larger and more complex templates, such as peptides and proteins, and even cells and viruses, among others. This tendency, exceptionally demonstrated in 2017 in the work of Culver and Peppas^17^ (see Figure 2), persisted until the present time.

**Figure 3:**
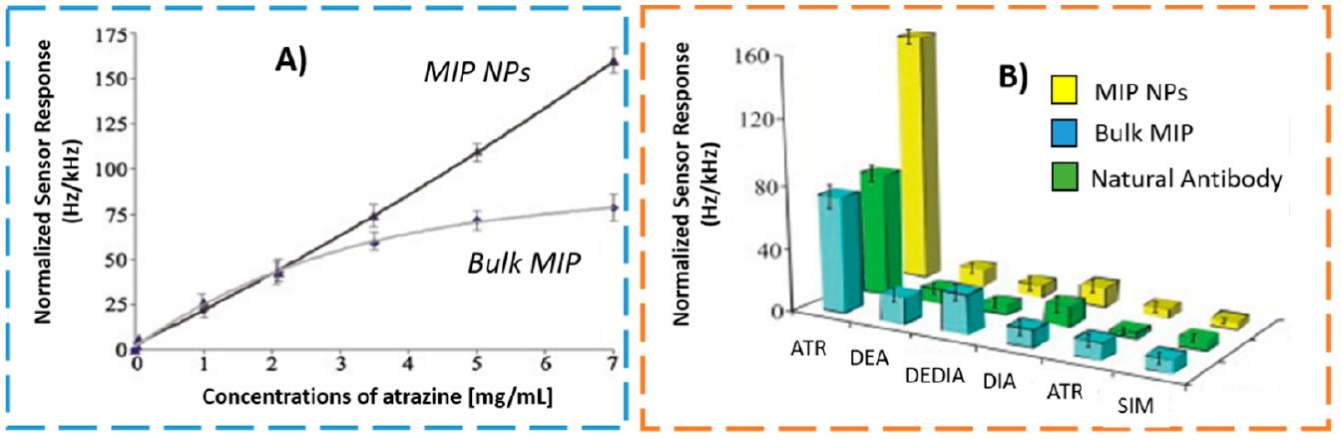
(A) Comparison of the piezoelectric sensor response to atrazine obtained by the MIP polymer layer and the MIP NPs; (B) sensor response obtained by natural and “plastic” antibodies to 7 mg L-1 atrazine and selectivity data against analogues molecules, namely des-ethyl atrazine (DEA), des-ethyl-des-isopropyl atrazine (DEDIA), des-isopropyl atrazine (DIA), propazine (PRO), and simazine (SIM). Adapted with permission from ref (18). Copyright 2011 Elsevier B.V.

The advantages in terms of biosensing performance of going from larger- to smaller-sized imprinted materials were reported by Yaqub et al.,^18^ in 2011, where the same set of acrylate/methacrylate monomers were used to prepare both MIP bulk polymeric layers and MIP NPs (45–85 nm) for specific recognition of atrazine. Then, the prepared MIP materials were incorporated into a piezoelectric sensor surface for comparison. As can be seen in Figure 3A, the sensor responded linearly to the concentration for the MIP NPs, while saturation was observed for the bulk MIP due to the high surface/volume ratio of NPs that increases the accessibility of the analyte to the receptor surface. In addition, the results obtained showed a more than 2-fold higher sensor response of imprinted NPs relative to the natural antibody with both showing a similar selectivity pattern relative to atrazine metabolites and structural analogues (Figure 3B). The size and uniform geometry of nanoMIPs leads to a low number of binding sites (ideally 1) per NP, reducing nonspecific binding (NSB) and providing faster kinetics of interaction.^19^ Overall, nanosized MIPs compete better with natural antibodies than bulk MIP materials in terms of size and affinity toward the target analyte, having the potential to substitute them as synthetic receptors in affinity assays and sensors for (bio)analysis of several (bio)compounds.^20,21^ To further empathize the merits of nanoMIPs, Table 2 provides the direct comparison of the detection levels (LOD) achieved in bioanalytical assays targeting the same (bio)molecule using synthetic and natural antibodies.

**Table 2:** Direct Comparison of LODs Reported for NanoMIPs-Based Assays vs Immunoassays

target	nanoMIP assay (LOD)	immunoassay (LOD)	observation	ref
vancomycin	ELISA-based assay (2.5 pM)	commercial ELISA (0.1 μM)	LOD of the nanoMIP-based assay was 3 orders of magnitude inferior	(22)
atrazine	QCM (20 ppb)	QCM (0.43 ppm)	NanoMIP assay with a LOD down to ppb	(18)
α-casein	SPR (0.127 ppm)	commercial ELISA (3 ppm)	LOD of the nanoMIP assay lower than LODs of commercial ELISA kits	(23)
cardiac troponin I	thermal detection (0.46 ng L^–1^)	several immunoassays (0.7–200 ng L^–1^)	LOD of the nanoMIP assay was inferior to most of immunoassays (Table S2)	(24)
SARS-CoV-2 (alpha variant)	thermal detection (9.9 fg mL^–1^)	thermal detection (8.9 fg mL^–1^)	LOD of the nanoMIP assay sensor was similar to the immunosensor	(25)

## Analyte-Responsive MIP Hydrogels

The fabrication of stimuli-responsive hydrogels is currently a rapidly expanding field that already found relevant applications in biomedicine and bioanalytical detection.^26,27^ The unique properties of hydrogels depend on the molecular arrangement of the 3D network of cross-linked polymer chains that changes in volume size (swelling or shrinking) in response to certain external physical (temperature, electric field, light, pressure, etc.), chemical (pH, solvent, ionic strength, etc.), and biological (proteins, nucleic acids, drugs, etc.) stimulus.^26^ The “swelling-behavior” of the gels arises from the ability of hydrophilic groups (−OH, −COOH, −NH_2_, etc.) composing the polymer structure to absorb and retain large amounts of water, providing the optimal environment for encapsulation of small molecules, proteins, and even cells. Furthermore, the possibility to properly synthesize hydrogels in different dimensions and configurations makes these functional materials very promising for the design of new “smart” biomaterials, such as MIPs.^28^

Molecularly imprinted hydrogels exploit the change in the swelling degree in response to molecular recognition of the target analyte.^29^ Despite the conformational changes of analyte-responsive hydrogels, the binding cavities remain stable and well-defined in the MIP structure, ensuring the recognition. Upon interaction with the analyte, the changes in volume and shape of MIP hydrogels also change its mechanical, electrical, or optical properties that can be used for signal transduction.^29^ Acrylic acid and acrylamide derivatives are by far the most employed backbone monomers for preparation of imprinted gels. Moreover, *N*-isopropylacrylamide (NIPAAm)^30^ has been of particular interest for production of high-quality MIPs. The polymer (PNIPAAm) has lower critical solution temperature (LCST) near 32 °C in aqueous solution which means that the polymer expands and contracts at temperatures below and above the LCST, respectively, altering its hydrophilicity/hydrophobicity equilibrium.

Over recent years, the developments in nanotechnology have paved the way for the production of hydrogel-based MIP NPs, i.e., imprinted nanogels. The mixture of NIPAAm with water-soluble polymerizable (meth)acrylates and (meth)acrylamides derivatives is one of the most popular approaches for synthesis of imprinted nanogels,^31^ providing high tunability of the framework for imprinting of large biomolecules in mild aqueous conditions.

## Routes for MIP NPs Synthesis

Over the years, many ingenious approaches have been reported for protein imprinting, aiming to obtain synthetic biomimetic materials of distinct formats, ranging from MIP thin films (prepared by deposition of self-assembled monolayers, electropolymerization, etc.), to membranes, or particles of different size and composition (surface imprinting of core–shell and metal NPs, for example), depending on its final application.^5,7,11,32^ In this review, we will focus on hydrogel imprinting approaches that are commonly employed for preparation of thermosensitive hydrogel-MIP NPs for recognition of several types of templates ranging from low molecular weight molecules to biomacromolecules.

**Figure 4:**
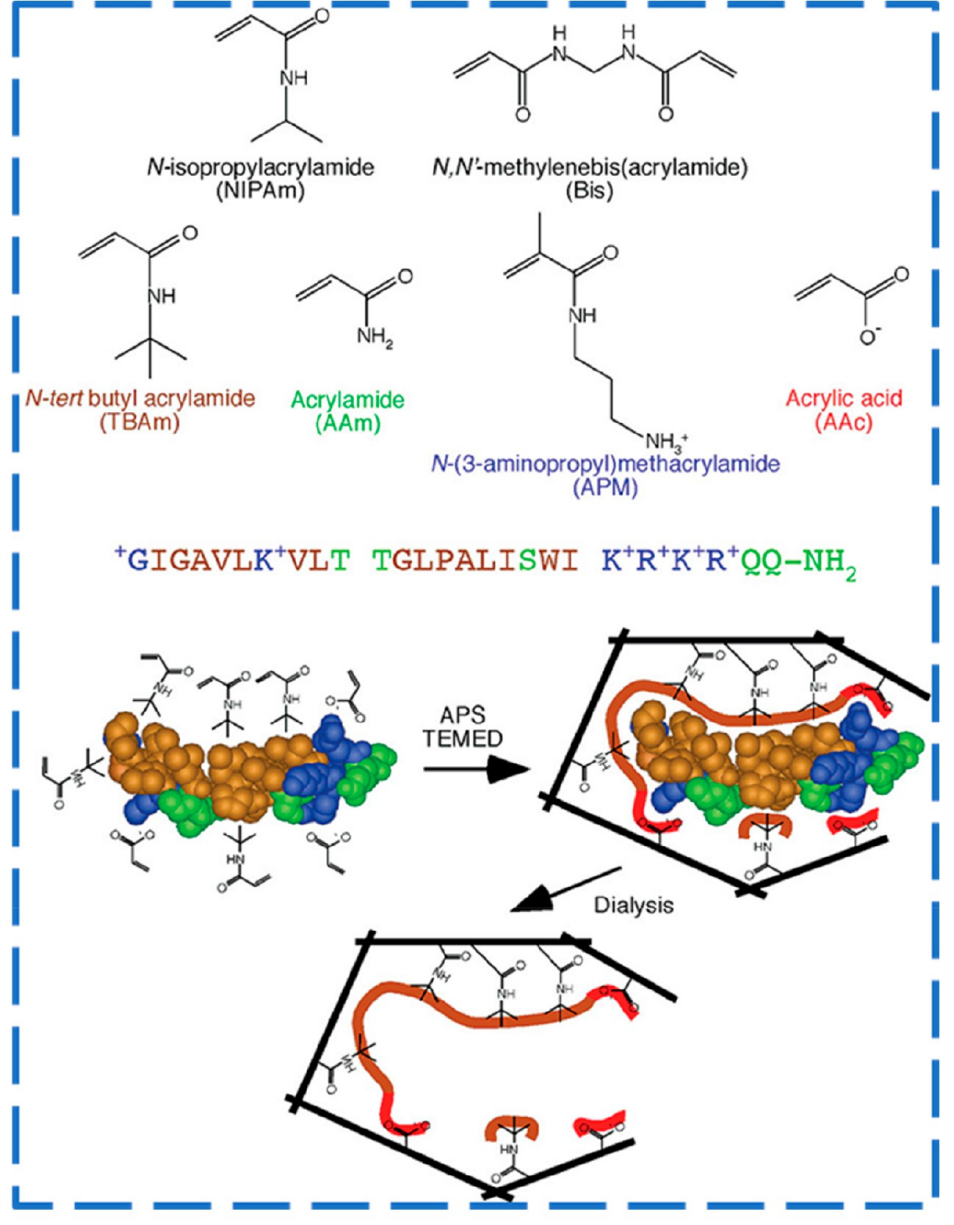
Schematic representation of monomers, template amino acids sequence (melittin), and imprinting process for NPs synthesis by precipitation polymerization. Reprinted from ref (31). Copyright 2008 American Chemical Society.

One simple approach for the preparation of MIP (micro/nano) particulates is the precipitation polymerization method that consists of performing cross-linking polymerization from a highly diluted solution containing a mixture of monomer(s), initiator, and template all dissolved in a suitable solvent. Under appropriate experimental conditions, monodisperse spherical polymer particles are formed within the reaction medium^20,21^ with diameter from a few micrometers^33^ to dozens of nanometers.^34^ However, many methods make use of organic solvents which are not suitable for proteins.^21^ Thus, a particularly attractive approach is to carry out precipitation polymerization in an aqueous-based environment (and in the presence of very low concentrations of surfactant)^35^ to obtain uniform spherical MIP NPs.^21^ The first work reporting the use of precipitation polymerization for routine one-step production of water-soluble MIP NPs was reported by Hoshino et al.,^31^ in 2008, where high-quality nanoMIPs were prepared for specific recognition of bee toxin melittin (Mel; see Figure 4). The concept was then extended for preparing MIP NPs with affinity for lysozyme,^36^ Fc fragment of IgG,^37^ fibrinogen,^38^ a vascular endothelial growth factor (VEGF165),^39^ among others.

**Figure 5:**
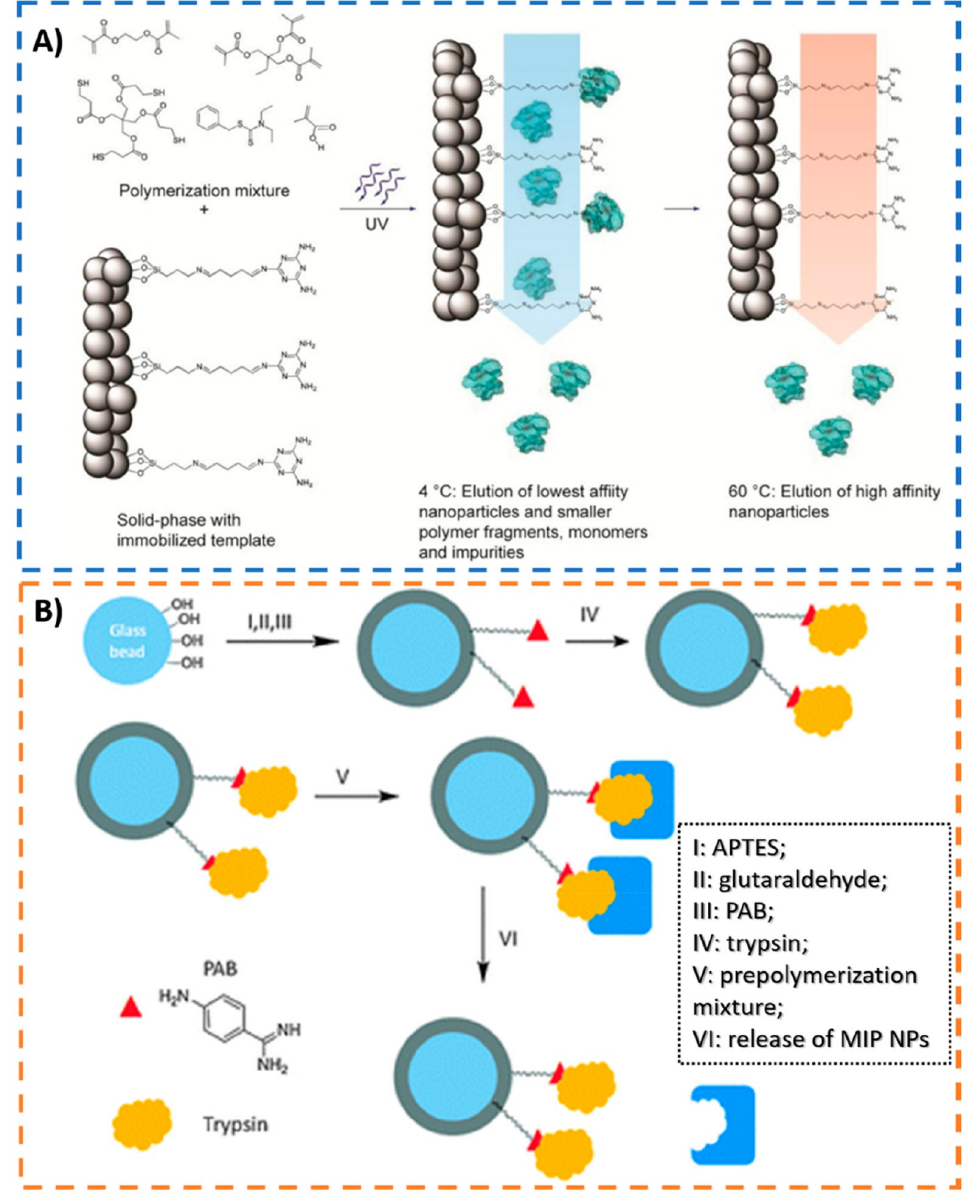
(A,B) Schematic representations of the synthesis process of MIP NPs by solid-phase imprinting for recognition of (A) small templates (for illustration purposes) and (B) large biomolecules. (A) Small templates were covalently bound to the glass beads, via 3-aminopropyltrimethyloxysilane (APTMS) and glutaraldehyde as linker, allowing its reusability. The polymerization was initiated by UV-irradiation. Adapted with permission from ref (40). Copyright 2013 John Wiley and Sons. (B) Affinity ligand *p*-aminobenzamidine (PAB) was used to reversibly immobilize the template protein and reload glass beads with protein if template degradation occurs. Redox initiator system ammonium persulfate (APS)/*N*,*N*,*N*′,*N*′-tetramethylethylenediamine (TEMED) was used for polymerization at 37 °C. Adapted with permission from ref (41). Copyright 2013 Royal Society of Chemistry.

Although precipitation polymerization is a very simple and efficient method to obtain MIP NPs in high yield and purity, the extraction of template is time-consuming (several days by dialysis), the synthesis makes use of surfactants unfavorable for biological applications, and the strength of monomer–template interactions can be reduced due to high binding site heterogeneity (highly polyclonal).^20,21^ To overcome these drawbacks, solid-phase synthesis (see Figure 5) has emerged in the literature as an attractive alternative method allowing one to obtain nanoMIPs resembling “monoclonal antibodies”. The first work in this context was reported by Poma et al.,^40^ in 2013, for imprinting of a low molecular weight template (melamine), vancomycin, and a model peptide (see Figure 5A), while Ambrosini et al.^41^ have extended the concept for protein recognition (trypsin) by performing aqueous polymerization under mild conditions (Figure 5B). In brief, the synthetic process is based on the immobilization of templates onto the surface of a solid support (usually glass beads) before polymerization aiming to reduce template’s degrees of freedom, thus yielding NPs with narrower affinity distribution. Furthermore, the exclusion of low-affinity MIP NPs is performed by simple solvent washing before collection of high-affinity nanoMIPs by thermoresponsive shrink (hot washing)^40^ or swelling (cold washing).^41^

High affinity nanoMIPs targeting small molecules,^42,43^ peptides,^44,45^ proteins,^44,46−48^ and also virus^25,49^ and bacteria^50^ have been prepared through well-established solid-phase synthesis protocols.^51^ In fact, this technology can benefit from automated synthesis for reproducible preparation of polymer batches^47^ and industrial mass production of MIP NPs.^20^ Still, the low yields of MIP NPs (<1 mg g^–1^ support beads) are perhaps the main limitation due to the low surface area of conventional glass beads (diameter of 70–100 μm), leading to low amounts of immobilized protein.^20,21^ Thus, alternative solid-phase materials have been attempted, such as (submicrometer-sized) magnetic carriers,^52,53^ whose high surface-to-volume ratio can improve the synthesis yield.

## Epitope Imprinting

**Figure 6:**
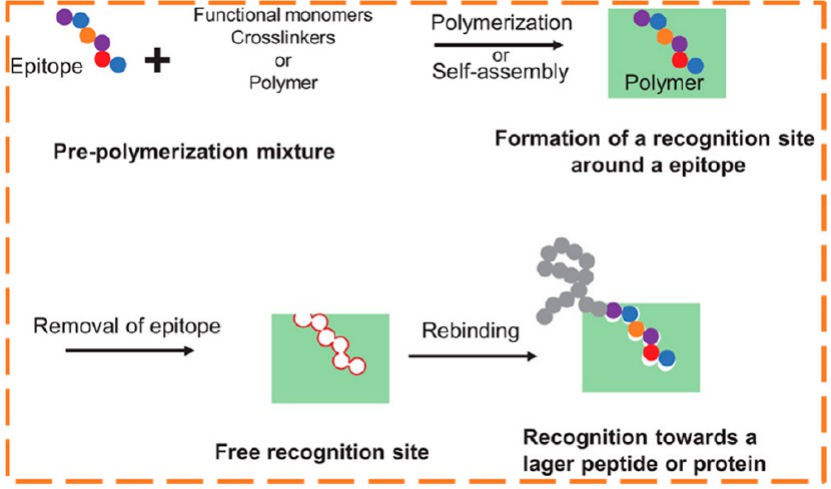
Schematic representation of epitope imprinting approach. Reprinted with permission from ref (55). Copyright 2019 John Wiley and Sons.

Although the MI of small molecules is already well-established through several approaches, the imprinting of proteins can be rather challenging due to its complex and flexible conformation that needs to be maintained during the polymerization process. Furthermore, the incorporation of the whole protein within the polymeric matrix can strongly inhibit the extraction procedure while some templates can be very expensive even for small quantities. To solve these issues, the epitope approach^54,55^ (see Figure 6) was introduced, bringing great advantages in terms of MIP affinity, performance, and production costs.

The epitope approach resembles antibody operation by recognizing only a small exposed fragment of the large protein (epitope; around 9 amino acids). For example, the bovine serum albumin (BSA) amino acids 599–607 (VVSTQTALA) and the cytochrome c (cyt c) amino acids 97–104 (AYLKKATNE) were selected as the unique imprinting peptides for specific MIP recognition. These terminal amino acids sequences were selected from the C-terminal region of the protein due to being less expected to undergo post-translational modifications.^56^ In the end, after epitope removal from the MIP matrix, the specific binding sites have the ability to recognize the entire target protein.^54,55,57^

Many protein epitopes are easily accessible from crystal structure data and epitope mapping. In addition to the contribution of molecular modeling for improved design of specific receptors by choosing the highest affinity monomers for a template,^58,59^ computational methods have been used for careful selection of suitable epitope(s) for protein templates.^60^

The use of epitopes is fully compatible with processes for MIP NP preparation.^48,61^ Epitopes bearing −NH_2_ or −COOH groups can be easily immobilized onto glass beads through well-established chemistry procedures, while cysteine groups can be added to the selected synthetic peptide (cysteine-epitope) for covalent attachment to solid supports using succinimidyl-iodo acetate as a linker.^62−64^

## Application of Hydrogel NanoMIPs for Biosensing of Macromolecules

### General Perspective

Nanosized MIPs have several potential applications due to their ability to selectively recognize and bind specific target molecules. A general overview of nanoMIPs applications is depicted in Table 3.

**Table 3:** Core Applications of MIP NPs Including Representative Examples for Which They Have Been Used

sample pretreatment and analytical separations	biomedical applications	biosensing
solid-phase extraction (SPE)^65,66^	bioimaging^62,64^	optical (colorimetric, surface plasmon resonance, fluorescence, etc.)
affinity chromatography^10^	drug delivery for cancer therapy^63,67^	electrochemical (potentiometric, amperometric, etc.)
capillary electrophoresis^68^	biomimetic nanomedicine^69^	other approaches (thermal detection, quartz crystal microbalance, etc.)

The preparation of imprinted materials for specific recognition of (bio)macromolecules is currently a hot topic since many peptides and proteins (i) act as important biomarkers of many prevalent diseases (cancer, infectious diseases, etc.), (ii) can induce allergic responses in susceptible individuals, or (iii) be a source of environmental pollution. As stated before, nanosized MIP particles lead to improved performance of sensing devices.^20,21^ Literature reviews focusing on MIP nanomaterials for chemical sensing are currently missing, considering the fast technological advances in this field. Particularly, this review aims to provide the current state of art of hydrogel based-MIP NPs sensors for detection of proteins (>1500 Da). Detection of pathogens based on imprinted nanogels for recognition of viral and bacterial proteins were also included in this review.

In order to provide a general overview on this topic, we start by summarizing the most representative works that emerged in the literature over the past decade, shown in Table 4, which includes the target biomolecule to detect, the synthesis approach used for nanoMIPs preparation, the sensor transduction mode, the strategy used for incorporating nanogels onto the sensing platforms, and the detection levels achieved (LOD).

**Table 4:** List of the Most Representative Works in the Literature Reporting the Integration of Hydrogel MIP NPs into Transduction Devices for Detection of Peptides and Proteins^a^

target biomolecule (sample medium)	synthesis approach	detection method	nanoMIPs immobilization	LOD/linear range	ref
vancomycin (blood plasma)	solid-phase imprinting	ELISA/MINA	adsorption by evaporation of MIP NPs solution overnight	2.5 pM/0.001–70 nM	(22)
vancomycin (blood plasma)	solid-phase imprinting	RI changes at an optical fiber LPG	covalent coupling of the nanoMIPs onto the fiber modified with APTMS and using GA as linker	10 nM/10 nM–700 μM	(75)
HAS (human serum)	epitope-mediated precipitation polymerization imprinting	fluorescence (using a hybrid MIP NPs-QDs composite)	–	44.3 nM/0.25–5 μM	(70)
hyaluronic acid (human keratinocytes)	epitope-mediated precipitation polymerization imprinting in DMSO	fluorescence imaging (using a polymerizable rhodamine derivative)	–	(*K*_D_ = 196 μM)	(74)
hepcidin-25	epitope-mediated precipitation polymerization imprinting	SPR	biotinylated MIP NPs anchored onto the SPR sensor chip with covalently immobilized NeutrAvidin	5 pM/7.2–720 pM	(71)
vancomycin	solid-phase imprinting	electrochemical (CV)	self-assembly of e-nanoMIPs on a Nafion membrane coated glassy carbon electrode	83 μM/83–410 μM	(45)
VEGF (zebrafish embryos)	epitope-mediated solid-phase imprinting	fluorescence imaging (using a hybrid MIP NPs-QDs composite)	–	(*K*_D_ = 1.56 ± 0.20 nM)	(62)
Trypsin (human serum)	solid-phase synthesis of core–shell MIP NPs	ELISA/fluorescence MINA (using FITC-labeled MIP NPs)	–	(LOQ = 50 pM)/50 pM–5 nM	(84)
EGFR; biotin; vancomycin; trypsin	epitope-mediated solid-phase imprinting	thermal detection	thermocouples functionalized with nanoMIPs by dip-coating	3–5 nM/0–100 nM; 0–500 nM	(44)
α-casein (CIP samples)	solid-phase imprinting	SPR	covalent attachment of the nanoMIPs to the carboxylated SPR chip through EDC/NHS coupling	0.127 ppm/0–150 ppm	(23)
trypsin; THC	solid-phase imprinting	electrochemical (capacitance)	covalent immobilization of nanoMIPs onto the electrode modified with electropolymerized tyramine film and using GA as linker	1.0 × 10^–14^ M/1.0 × 10^–14^–1.0 × 10^–9^ M; 1.0 × 10–14 M/1.0 × 10^–12^–1.0 × 10^–5^ M	(81)
EGFR (MCF-7 breast cancer cells)	protein-imprinted poly(NIPAAm) SAM on the surface of Au nanorods	Raman imaging (using SERS-active MIP nanoprobes)	–	(≥10^–14^ mol L^–1^)	(83)
mycobacterium leprae bacteria (human blood)	epitope-mediated radical polymerization imprinting	electrochemical QCM (EQCM)	incorporation of the nanoMIPs on the EQCM transducer by electrochemical polymerization of 4-ATP	0.161 nM/10–140 nM	(50)
insulin (human plasma)	epitope-mediated solid-phase imprinting	electrochemical (DPV)	covalent immobilization of the e-nanoMIPs onto the SPE via APTES and using GA as linker	81 fM/50–2000 pM	(79)
leukotrienes; insulin (urine/plasma)	solid-phase imprinting	ELISA/fluorescence MINA in magnetic microplates	–	0.73 pM/0.45–364 pM; 27 pM/25–2500 pM	(77)
human transferrin (human serum)	precipitation polymerization imprinting	POF-SPR	soft nanoMIPs covalently coupled to the carboxylated plasmonic surface by EDC/NHS reaction	1.2 fM/1.2 fM–1.8 pM	(72)
glucose; paracetamol; C4-HSL; THC; trypsin (spiked plasma)	solid-phase imprinting	electrochemical (DPV)	covalent immobilization of e-nanoMIPs to the SPE previously modified with cysteamine SAM followed by EDC/NHS reaction	0.43 mM/0.8–50 mM; 82 μM/100–1000 μM; 0.12 nM/6.25–800 nM; 0.05 μM/0.1–1000 μM; 0.20 nM/6.5–100 nM	(80)
troponin I	epitope-mediated solid-phase imprinting	thermal detection	nanoMIPs covalently coupled to the SPE surface via electrografting of 4-ABA and EDC/NHS reaction	0.46 ng L^–1^/0–2 ng L^–1^	(24)
SARS-CoV-2 virus (samples from COVID-19 patients)	epitope-mediated solid-phase imprinting	thermal detection	nanoMIPs covalently coupled to the SPE surface via electrografting of 4-ABA and EDC/NHS reaction	9.9 fg mL^–1^, alpha variant 6.1 fg mL^–1^, delta variant/1 fg mL^–1^–10 pg mL^–1^	(25)

a MINA: molecularly imprinted polymer nanoparticle-based assay; RI: refractive index; HSA: human serum albumin; LPG: long period grating; APTMS: 3-aminopropyltrimethyloxysilane; GA: glutaraldehyde; QDs: quantum dots; DMSO: dimethyl sulfoxide; CV: cyclic voltammetry; VEGF: vascular endothelial growth factor; FITC: fluorescein isothiocyanate; EGFR: epidermal growth factor receptor; CIP: cleaning in place; SAM: self-assembled monolayer; EDC: 1-ethyl-3-(3-(dimethylamino)propyl)carbodiimide; NHS: *N*-hydroxysuccinimide; THC: tetrahydrocanabinol; 4-ATP: 4-aminothiophenol; DPV: differential pulse voltammetry; SPE: screen-printed electrode; APTES: (3-aminopropyl)triethoxysilane; POF: plastic optical fiber; C4-HSL: C4-homoserine lactone; 4-ABA: 4-aminobenzoic acid.

Hydrogel MIP NPs targeting analytes ranging from small peptides, such as vancomycin, to high-molecular-weight proteins (human serum albumin, human transferrin, epidermal growth factor receptor, etc.) were successfully produced. Some works used the precipitation polymerization^70−74^ method for simple one-step preparation of biomimetic materials. However, the increasing popularity of the solid-phase approach^22−25,44,45,62,66,75−81^ is remarkable due to its ability to produce MIP materials resembling monoclonal antibodies. Interestingly, several works reported the advantages of epitope imprinting^24,50,62,66,70,71,74,79^ to obtain good quality nanoMIPs. Straightforward bioinformatic methods showed their great potential for proper epitope identification. For example, a short peptide fragment (∼10 amino acids) from the receptor binding domain (RBD) of the spike protein of SARS-CoV-2 was identified by *in silico* analysis and used as the epitope of the target virus.^25^ In another work, insulin epitopes were identified using *in silico* epitope mapping while the nanogel composition was computationally designed to selectively bind selected epitopes.^79^ Simple and efficient immobilization of SH-cysteine modified epitopes onto the solid supports was achieved throughout covalent coupling.^44^

The development of new surface modification procedures^82^ or the incorporation of labels, such as electroactive probes^45^ and fluorophores,^74,77^ into the MIP hydrogel structure by simple adjustment of the monomer composition greatly contributed to improvements on MIP-based sensing methods. For preparation of the sensing devices, nanoMIPs were easily integrated into detection platforms through simple (i) physisorption,^22,44,76^ using (ii) biotin-neutravidin mediated immobilization,^71^ by (iii) incorporation into electropolymerized polymers^50^ and via (iv) covalent attachment.^23,24,72,73,75,78−81^ From those, the covalent coupling of synthetic receptors to substrates showed to be the most versatile and efficient immobilization procedure for stable and reproducible sensor response.^24^

The applicability of sensing devices in real (or close-to-real) conditions is fundamental to envisage a practical application. Although some works focused on proving that the sensing concept is feasible by performing detection studies in buffer solution, other sensors succeeded when operating in complex samples, such as human urine^77^ and serum/plasma^22,70,72,75,79,80^ and even real patient samples (blood,^50^ respiratory tract,^25^ etc.). Still, more applied work of sensors operating directly in complex samples is needed to demonstrate the potential of the biomimetic devices. The sample preparation should be minimal (only dilution, if possible) to make the sensing devices easy-to-use.

It is important to notice that electrochemical and thermal methods have already started transitioning from the lab to the field by using disposable and cost-effective screen-printed electrodes (SPEs)^24,25,79,80^ as substrates for detection, enabling mass production of MIP-sensor devices. This opened routes for the point-of-care (POC) detection of proteins (and pathogens), enabling the early disease diagnosis and/or the monitoring of patient health at the site of care.

Overall, methods incorporating imprinted nanogels for protein biosensing mainly relied on: (i) optical sensors, such as ELISA-based assays,^22^ surface plasmon resonance (SPR),^23,71^ surface-enhanced Raman spectroscopy (SERS)^83^ and fluorescence sensors;^62,70^ (ii) electrochemical sensors, based on either capacitance^81^ or amperometric measurements;^80^ and (iii) other sensing approaches, such as thermal^44^ and piezoelectric methods.^50^

In the following sections, we discuss the main achievements of this technology from several perspectives, such as nanogels composition and preparation, immobilization of artificial receptors on biosensing platforms, detection levels achieved, setup design (simplicity, cost, portability, sample volume, etc.), and applicability of developed sensor devices.

### Optical Sensors Based on Hydrogel Imprinted NPs

The enzyme-linked immunosorbent assay (ELISA) is a conventional method for detection and quantification of proteins in several fields, ranging from fundamental research to biomedical applications,^85^ such as the early disease diagnosis where measurement of ultralow protein concentration levels are needed.^85,86^

**Figure 7:**
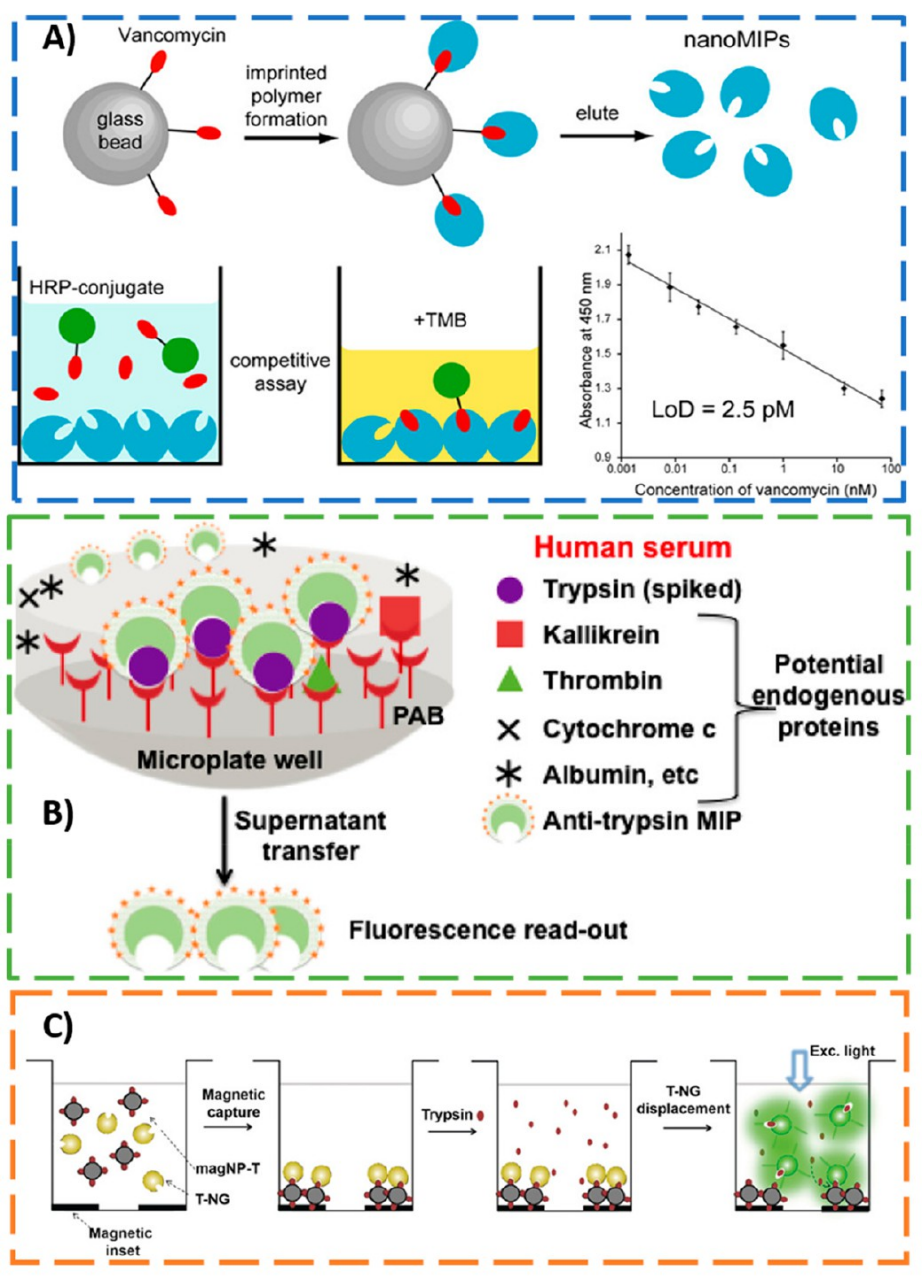
(A) Schematic representations of the (i) MIP NPs preparation by a solid-phase approach, (ii) developed nanoMIP-based ELISA assay for detection of vancomycin, and (iii) calibration curve obtained. Adapted from ref (22). Copyright 2013 American Chemical Society. (B) Illustration of the sandwich immunoassay for trypsin detection in human serum using fluorescent core–shell MIP NPs and *p*-aminobenzamidine (PAB)-functionalized plates. Adapted from ref (84). Copyright 2017 American Chemical Society. (C) Schematic representation of the magnetic template-based fluorescence competitive displacement assay for detection of trypsin and pepsin. Adapted with permission from ref (53). Copyright 2019 John Wiley and Sons.

One strategy to achieve high assay sensitivity is to increase the binding capacity of the capture (bio)receptors. This principle was explored by Yonamine et al.^87^ and Chianella et al.^22^ that engineered nanoMIPs as substitutes for natural receptors in standard ELISA assays. These ELISA-like assays are commonly referred to in the literature as MINA: molecularly imprinted nanoparticle-based assay.^88^ To develop the ELISA-based assay for glycopeptide antibiotic vancomycin, the nanoMIPs, prepared by the solid-phase approach, were first placed in microplate wells by simple evaporation. Then, a competition/inhibition assay format between vancomycin and horseradish peroxidase (HRP)-vancomycin conjugate was employed using 3,3′,5,5′-tetramethylbenzidine (TMB) as a chromogenic substrate for color development (see Figure 7A). After optimization of blocking and washing protocols, a linear logarithmic calibration curve between 1 pM and 70 nM was obtained. The LOD obtained (LOD = 2.5 pM) by the MINA was 3 orders of magnitude inferior to traditional ELISA (0.1 μM), probably due to the higher binding capacity of MIP NPs, leading to the enrichment of the sensing platforms with a target analyte. Furthermore, the artificial receptors provided high assay selectivity for vancomycin against other antibiotics, and the MINA applicability was demonstrated by detection of vancomycin in complex blood plasma. The high assay sensitivity allowed a 100,000-fold dilution of spiked plasma samples which greatly contributed to minimize the interference of the serum matrix.

Given the possibility of custom synthesis of MIP NPs, the MINA concept was further adapted for detection of enzymes (HRP, cytochrome C)^76^ and mycotoxins.^89^ These pioneer works were very inspiring for more applied work in ELISA-based colorimetric assays. Besides, the inherent stability and low cost production of MIP NPs offer economic benefits from storage and transportation point-of-views since no refrigeration of MINA kits is required.

Meanwhile, the advances in fluorophore labeling chemistry brought in innovative features to MINA.^53,84^ In 2017, Xu et al.^84^ developed core–shell MIP NPs for fluorescence quantification of trypsin (see Figure 7B) in undiluted human serum. The core–shell MIPs were prepared by a multistep solid-phase approach with postfunctionalization with fluorescein isothiocyanate (FITC) on the surface of the shell. The nanoMIPs showed good selectivity for trypsin against endogenous serine proteases (kallikrein, thrombin) and serum proteins (human serum albumin and cytochrome C), and a low limit of quantification (of 50 pM) was obtained.

Recently, in 2019, protein-modified magnetic NPs were combined with fluorescent-labeled nanoMIPs as reporters in the same competitive displacement assay to achieve ultrasensitive detection levels (see Figure 7C). The developed method is performed in magnetic microplates in a label-free manner, thus without the need for ligand or nanoMIPs immobilizations or enzyme conjugations.^53^ The generic fluorescence MINA approach was then applied for detection of leukotrienes (LTE4) and insulin in biological samples (urine and plasma) at physiological levels (LODs: 0.24–27 pM).^77^ Overall, the high sensitivity of fluorescence MINA is comparable to chromatographic techniques showing great potential for protein biomarker detection in a clinical diagnosis context.

Fluorescence biosensors have been facing tremendous progress with several relevant applications in bioanalysis, imaging of biological processes, measurement of molecular dynamics in cells, among others.^85,90^ On the other hand, hydrogel MIP NPs already showed their ability to cross biological membranes without toxicological effects on cells or living animals.^91,92^ The merge between MI nanotechnology and fluorescence sensing can be considered relatively simple. Fluorescence nanoMIPs can be prepared by adding an acrylate monomer bearing a fluorophore (fluorescein o-acrylate, methacryloxyethyl thiocarbamoyl rhodamine, *N*-fluoresceinylacrylamide, etc.) to the polymerization solution.^53,63,64,74,77,92^ Alternatively, quantum dots (QDs) and carbon dots (CDs) can be coupled to nanoMIPs to develop hybrid affinity nanomaterials for fluorescence protein quantification^70,93^ or bioimaging.

Haupt and his collaborators, in 2015, were the first to demonstrate that fluorescence nanoMIPs can be used for cell and tissue imaging by localizing and quantifying target biomolecules (hyaluronic acid) on cells.^74^ After this seminal work, fluorescence imaging was further applied for the *in vivo* study of (i) epidermal growth factor receptor (EGFR) in breast cancer cell lines,^63^ (ii) hyaluronic acid (HA) in human skin keratinocytes,^94^ and (iii) β2 microglobulin (B2M) as a means to detect senescent cells in mice.^64^

**Figure 8:**
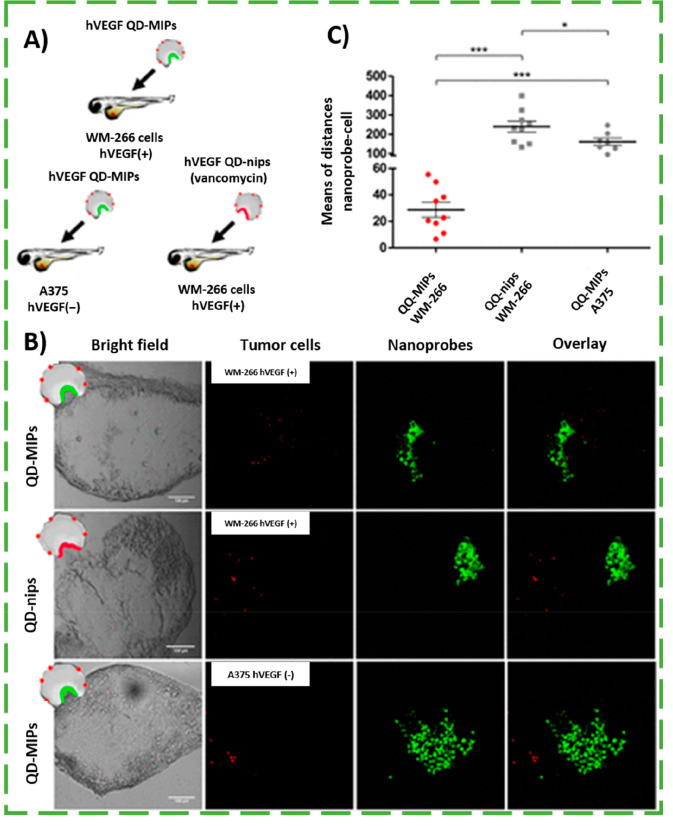
(A) Schematic representation of the *in vivo* experiments performed in zebrafish embryos as animal model where the hybrid fluorescence nanoMIPs (QD-MIPs) were injected in the hVEGF(+) model (WM-266) and in the hVEGF(−) model (A-375). Control experiments with fluorescence nanoMIPs against vancomycin (QD-nips) were injected in the hVEGF(+) model (WM-266). (B) Confocal bright field and fluorescence images along with the overlay signals of the *in vivo* experiments. (C) Statistical analysis of the mean of distances nanoprobe–cell (μm) for the three experiments performed (*p*-value = 0.0006; embryos *n* ≥ 7). Adapted from ref (62). Copyright 2017 American Chemical Society.

In another work, Cecchini et al.^62^ monitored the *in vivo* cellular dynamics of vascular endothelial growth factor (VEGF) which is overexpressed in many invasive cancers. For fluorescence imaging, nanoMIPs targeting human VEGF (hVEGF) were then covalently coupled with CdTe QDs. To test the ability of the fluorescence hybrid nanoMIPs for measuring the changes of hVEGF expression in cancer cells, two tumor models were obtained by injecting two human malignant melanoma cell lines in transparent zebrafish embryos, one with overexpressed hVEGF [WM-266, hVEGF(+)] and the other with low expression of hVEGF [A-375, hVEGF(−)] (see Figure 8A). After incubation with the nanoprobes for 7 h, the collected bright field and corresponding confocal microscopy images of tested models indicated that the fluorescence nanoMIPs were able to distinguish cells overexpressing hVEGF [hVEGF(+)] from noncancer cells [hVEGF(−)], being located close to the tumor mass (see Figure 8B). By opposition, the control QD-nips were not able to localize the tumor mass. In addition, the statistical treatment of the nanoprobe–cell distances further supported the experimental observations (Figure 8C), confirming the ability of QD-MIPs to selectively recognize tumor cells. Importantly, the prepared fluorescence nanoMIPs induced negligible toxic effects to zebrafish embryos. These studies highlight the great potential of fluorescence imprinted nanogels as imaging tools for cancer diagnostics and/or patient’s follow-up.

Plasmonic biosensors are a very popular method for real-time monitoring of protein binding events^95^ by measuring the refractive index (RI) changes occurring very near a thin metal film surface.^96^

**Figure 9:**
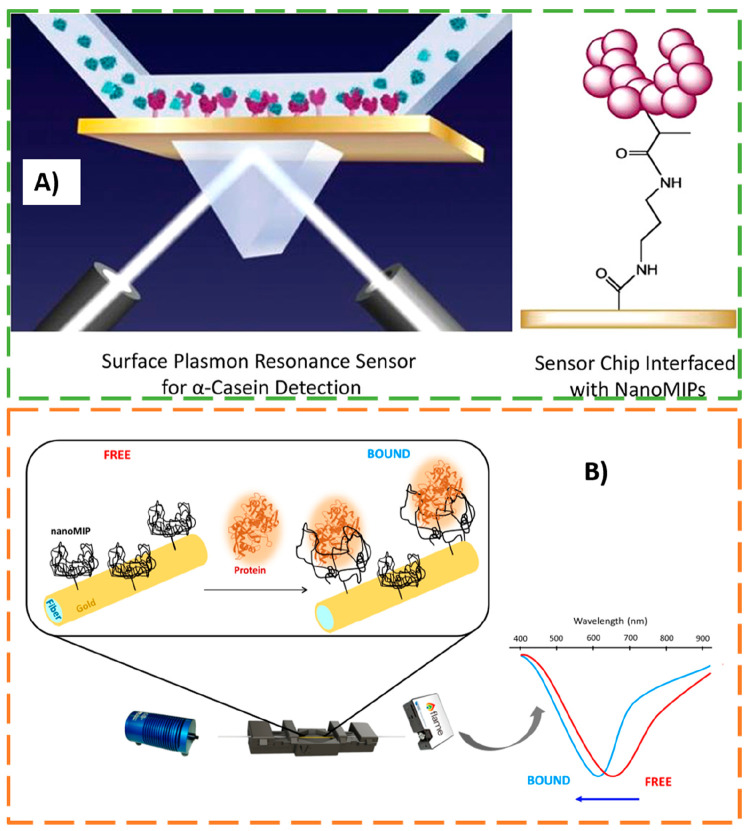
(A) General schematic representation of the nanoMIPs-SPR-based sensor for detection of milk allergen α-casein. Adapted from ref (23). Copyright 2018 American Chemical Society. (B) SPR sensor composed of a silica light-diffusing fiber (LDF) functionalized with soft nanoMIPs as artificial receptors for specific detection of human serum transferrin (HTR). Adapted with permission from ref (73). Copyright 2022 MDPI.

Recently, Tothill and collaborators^23^ reported the integration of nanoMIPs in SPR sensors for detection of milk allergen bovine α-casein. The sensor was fabricated to act as a simple and cost-effective (online or at-line) tool in the food manufacturing industry for routine monitoring of wash samples from cleaning in place systems (CIP). For α-casein detection, nanoMIPs with high selectivity for target allergen were covalently attached to the gold SPR chips (see Figure 9A) followed by cumulative surface injections of allergen protein solutions (from 0–150 ppm). The estimated detection limit (LOD of 127 ± 97.6 ng mL^–1^; 0.127 ppm) was even inferior to the LODs obtained by commercial ELISA kits. The SPR sensor showed a high selectivity for target α-casein in the presence of β-lactoglobulin (BLG) and BSA as matrix interferents, and satisfactory recovery values (87–120%) were obtained for α-casein-spiked CIP wash samples, after sample treatment by gel filtration. Furthermore, the same approach was employed for detection of milk protein β-lactoglobulin (BLG),^78^ bacterial endotoxins,^58^ and pathogenic viruses.^97^ Although the plasmonic sensors showed a high sensitivity (LODs of few ng mL^–1^), detection studies in close-to-real conditions are missing. Nevertheless, the SPR method integrating nanoMIPs showed its high potential for selective, rapid, and sensitive real-time monitoring of allergens and/or contaminants, helping to mitigate risks for consumers.

In another work, the significant RI changes resulting from conformational alterations of analyte-responsive MIP nanogels were used for the first time for plasmonic sensing.^72,73^ The shrinking and swelling of the nanoMIPs coupled to the tested fibers after binding to the target protein led to a blueshift of the plasmonic minimum (see Figure 9B). Human serum transferrin (HTR) was used as a model protein. The soft nanoMIPs composition was optimized to ensure polymer mechanical rigidity by using high cross-linking density (80% mol/mol of cross-linker). Ultrasensitive detection of HTR (LODs of few femtomolar) was achieved using both plastic optical fiber (POF)^72^ and silica light-diffusing fiber (LDF)^73^ plasmonic platforms functionalized with the soft nanogels. Although the sensing systems were successfully applied for detection of HTR in (10^6^ times) diluted serum mimic and human serum,^72^ more applied work of developed plasmonic devices operating in complex biofluids is needed. Still, these works demonstrated that soft nanoMIPs can be promising receptors for improved protein analysis by active plasmonics^72,73,98^ or MALDI-TOF-MS^99^ and can bring in new opportunities to the MIP biosensing field.

**Figure 10:**
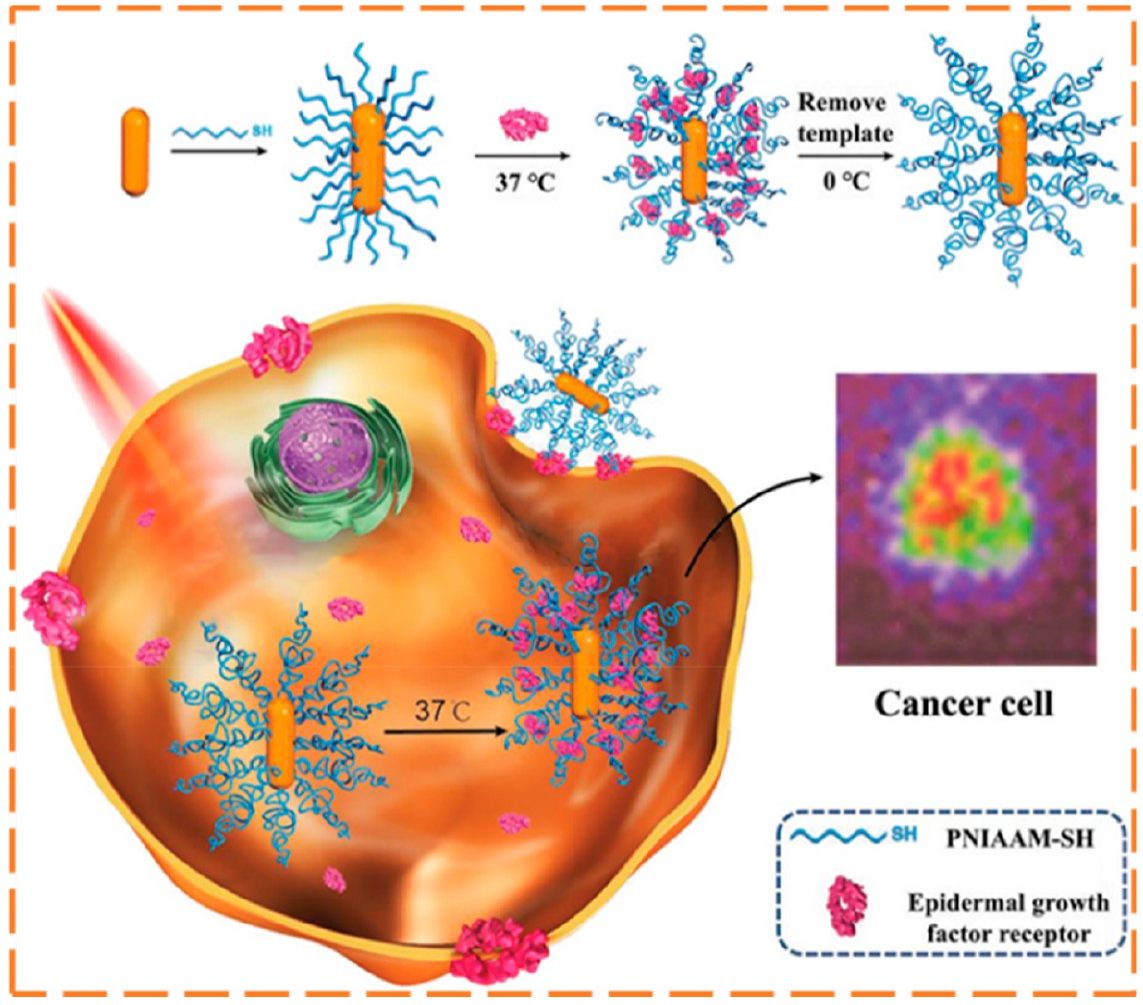
Schematic representation of the Raman imaging of epidermal growth factor receptor (EGFR) biomarker within live cells using SERS nanotags, consisting of gold nanorods coated with protein-imprinted poly(*N*-isopropylacrylamide) layers, whose responsive behavior is triggered by alteration of external temperature (also allowing nanoprobe recycling). Reprinted with permission from ref (83). Copyright 2018 John Wiley and Sons.

The scientific community is currently making efforts to come up with new synthetic procedures to prepare nanocomposites with efficient Raman enhancement.^100^ Thus, the combination of the selectivity of MIP materials with the large plasmonic enhancement of Au NPs can be very attractive for the development of new MIP-based SERS sensors.^101^ In this context, Zhang et al.,^83^ in 2018, developed a Raman imaging tool for monitoring cancer biomarkers in live cells based on biocompatible Au NPs coated with a thermal-responsive imprinted hydrogel as SERS nanoprobes. The nanocomposite was used for intracellular Raman visualization of epidermal growth factor receptor (EGFR) that is overexpressed in MCF-7 breast cancer cells. For obtaining the SERS nanotags, reversible addition–fragmentation chain transfer (RAFT) polymerization was used for deposition of a homogeneous MIP self-assembled monolayer (SAM) of thiolated poly(*N*-isopropylacrylamide) on the surface of gold nanorods as inorganic cores (see Figure 10). After extraction of template protein from the imprinted layer, the responsiveness of poly(NIPAAm) to the external temperature was used for capture (at 37 °C) and release (at 0 °C) of the target biomarker from the imprinting cavities due to the changes in the hydrogel structure, thereby altering the Raman signals enhance by the gold nanorods (see Figure 10). Ultimately, this pioneer work showed that intelligent SERS-active imprinted nanogels can be very promising for label-free cell imaging in the clinical field for disease diagnosis.

### Electrochemical Sensors Based on Hydrogel Imprinted NPs

Over recent years, the field of electrochemical biosensing faced tremendous growth due to the unique features of electrochemical methods, such as high sensitivity and reproducibility, fast response time, and cost-effective detection.^102,103^ Further, the advances in microelectronics and microfabrication allowed the portability of sensing devices for POC testing and/or *in situ* monitoring of a wide range of analytes.^104,105^ Thus, it is not surprising the large number of recent reviews highlighting relevant applications of electrochemical sensing for early disease diagnosis,^102,105,106^ drug monitoring,^107^ food quality and safety,^13^ environmental analysis,^12^ illicit drugs detection,^103^ among others. Moreover, MIP biomimetic materials have been incorporated into detection devices for electrochemical sensing through different methods of analysis (amperometry, potentiometry, and impedance).^5,6,12,13,108^

Amperometric biosensors can be divided into two main groups depending on the nature of the template: electroactive^109^ or nonelectroactive.^110^ MIP assays for detection of nonelectroactive biomolecules (peptides, proteins, etc.) are based on indirect electrochemical measurements performed in the presence of an external biocompatible redox probe (ferrocyanide/ferricyanide redox couple, etc.),^110^ using cyclic voltammetry, differential pulse voltammetry, etc. The quantification is based on the so-called gate effect,^111^ meaning that the binding of target analyte to MIP cavities alters the diffusional behavior of the probe through the receptor film. In order to eliminate the need of secondary diffusional process, an innovative approach emerged in 2014 by Udomsap et al.^112^ with the introduction of the electrochemical MIP (e-MIP). The e-MIP was prepared by adding an electroactive functional monomer (vinylferrocene, VFc) to the polymerization mixture, making the cross-linked MIP suitable for dual function of recognition unit and redox reporting system. As proof of concept, a polycyclic aromatic hydrocarbon (PAH) was used as the model template whose specific interaction with MIP particles tagged with VFc probes caused detectable changes in the ferrocene redox signals. Overall, the e-MIP microparticles (of size from 1.5 to 2.4 μm) were shown to be versatile receptors for direct (one-step) amperometric quantification of both small molecules^112,113^ and macromolecules.

**Figure 11:**
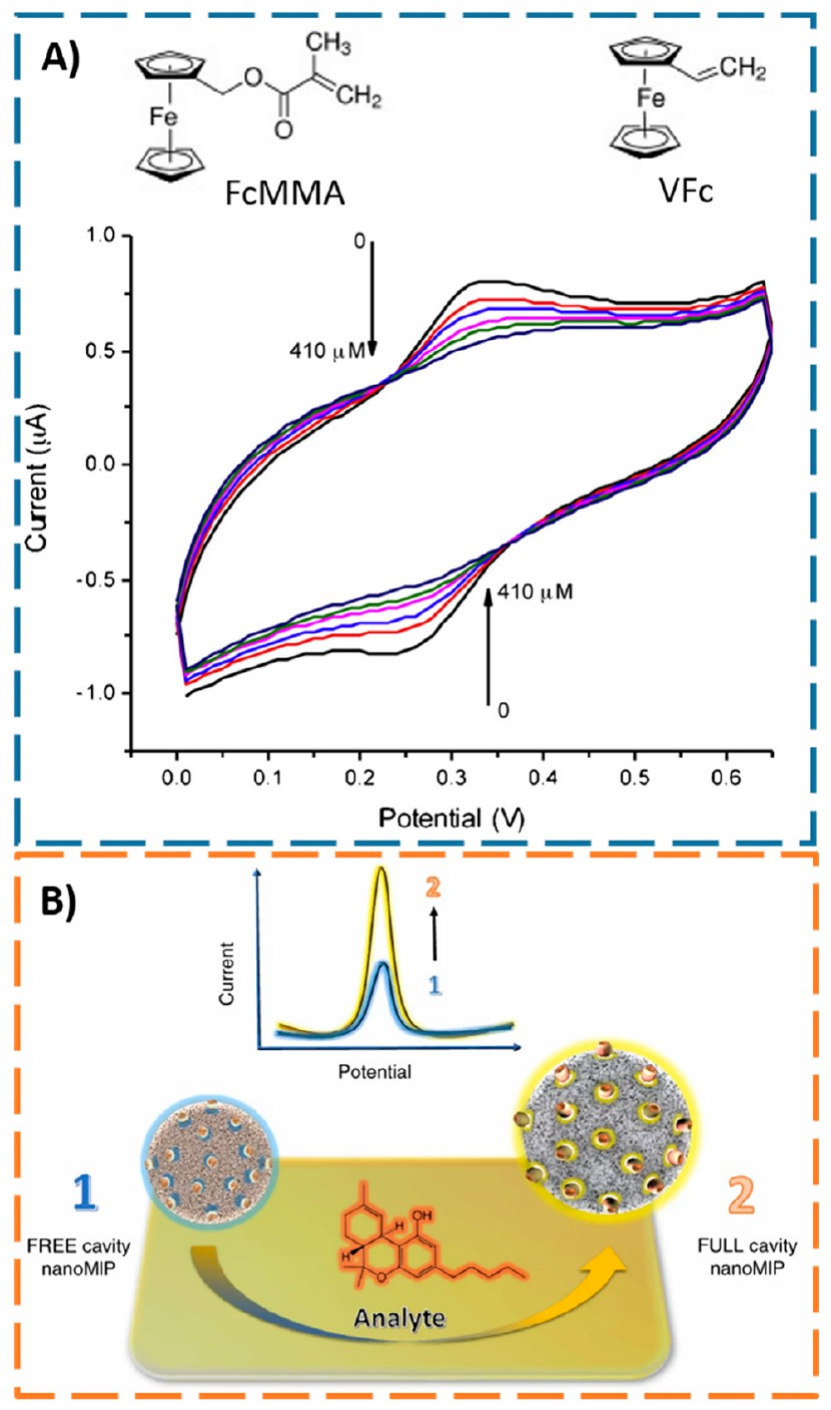
(A) Schematic representations of electroactive functional monomers ferrocenylmethyl methacrylate (FcMMA) and vinylferrocene (VFc); CV response recorded by the e-nanoMIPs modified electrode to increasing vancomycin concentration. Adapted with permission from ref (45). Copyright 2016 Elsevier B.V. (B) Generic schematic representation of the electroactive MIP NPs (e-nanoMIPs) combining selective analyte recognition and acting as redox reporting system due to changes of the polymer conformation after target analyte binding. Adapted with permission from ref (80). Copyright 2020 Springer Nature.

Triggered by the dual properties of e-MIP particles, but going down on size, Mazzotta et al.^45^ synthesized for the first time nanosized electroactive MIPs (e-nanoMIPs) by adding different amounts of two ferrocene-derivative monomers (see Figure 11A), vinylferrocene (VFc) and ferrocenylmethyl methacrylate (FcMMA), to the polymerization medium. After CV characterization to evaluate the monomers electroactivity and reversibility, the FMMA monomer (3%) was selected for the e-nanoMIPs preparation targeting vancomycin. The e-nanoMIPs were then deposited by self-assembly on a Nafion membrane-coated glassy carbon electrode. The electrochemical detection was based on the interaction of vancomycin with the ferrocene moiety that incrementally hindered the CV electron transfer process with the increasing analyte concentration (see Figure 11A). Although detection of vancomycin in biofluids is missing in their work to test the applicability of the electrochemical sensor in clinical context, the conceptual use of MIP NPs incorporating redox traces for direct amperometric sensing of large molecules was fully demonstrated. In another work, the same approach was used to develop an amperometric sensor for insulin detection.^79^ Computational modeling tools were first used to identify surface peptides (epitopes) as templates for imprinting and to design the multicomponent polymeric mixture. NanoMIPs having different chemical composition were synthesized, and the experimental conditions for covalent attachment to the screen-printed electrode (SPE) surface were optimized to enhance the sensor response. Good analytical features were found for the electrochemical chips, incorporating best performing MIP NPs, such as high reproducibility and sensitivity (LOD of 21 fM). Importantly, the sensor response to selected interferents (hemoglobin, human serum albumin, and human proinsulin C-peptide) was relatively low (<22%). The amperometric sensor was able to quantify insulin at clinically relevant levels in spiked human plasma samples (LOD of 81 fM), confirming its potential for POC monitoring of insulin in patients. Moreover, long-term stability studies showed a sensor response decrease (for 500 pM of insulin) of 35% over 168 days, revealing fair storage stability (at 4 °C) and robustness.

After these pioneer works highlighting the technological advances of amperometric sensors employing e-nanoMIPs, efforts were made by the research team headed by Professor Sergey Piletsky at the University of Leicester (UK) toward the production of generic amperometric devices not only for POC clinical diagnosis but also for *in situ* forensic, environmental, and food monitoring applications, by targeting both small molecular targets and also proteins in biological fluids (spiked plasma).^80^ The electroactive nanoreceptors were covalently immobilized at the surface of low cost and disposable screen-printed electrodes (SPEs), enabling the mass production of user-friendly electrochemical devices. Relative to the role of the hydrophilic nanogels in the bioanalytical assay, the analyte binding event induces changes in the hydrogel conformation that alters the exposed ferrocene probes attached to the MIP surface, thus favoring (or inhibiting) the electron transfer process (see Figure 11B) that can be easily monitored by common electrochemical techniques. The fabricated disposable chips had a shelf life of at least 6 weeks (at 25 ± 2 °C and relative humidity of 60 ± 5%).

One possible limitation of this technology is the relatively small faradaic currents arising from the self-reporting e-nanoMIPs. However, the increase of molar content (to 15–25%) of ferrocene-monomer (FcMMA)^114^ in polymer composition can enhance detection sensitivity. From a general perspective, the redox signals arising from electrochemical nanoMIPs can be further improved by modifying the bare electrode surface with electrocatalytic nanomaterials (or nanocomposites), such as metal NPs, graphene, carbon nanotubes, QDs, etc.

Potentiometric sensors can be a very attractive analytical tool due to their fast response, simple operation, portability, low cost, and wide working dynamic range.^115^ Although works combining electrochemical sensing and MIP NPs as artificial receptors for potentiometric detection of small molecules^43,116^ can be found in the literature, as far as we know, no potentiometric method incorporating MIP NPs for screening large molecules were reported so far. This is probably due to issues related to proteins electrical charge and structure flexibility. Although they have a net charge whose magnitude depends on its isoelectric point and the pH of the coupling buffer, proteins possess multiple charge locations that change according to their conformation,^117^ possibly affecting the stability of potentiometric measurements and/or requiring exhaustive optimization of experimental conditions to observe changes in the MIP surface potential.

Similarly to potentiometric sensors, impedimetric/capacitive sensors employing MIP NPs are currently scarce.^81,118^ Still, Canfarotta et al.^81^ showed that these sensing systems can be very useful for label-free detection of low levels of analyte. In their work, capacitive measurements allowed the sensitive detection (LODs of 1.0 × 10^–14^ M) of two molecules having very different molecular weights, tetrahydrocannabinol (THC) and trypsin. To attach the artificial receptors to the electrode surface, a poly(tyramine) film was first deposited on the electrode surface by electropolymerization followed by covalent coupling using glutaraldehyde (GA) as a linker. Although the electrochemical sensor was shown to be suitable for quantification of physiologically levels of target analytes, studies of the sensor operating in close-to-real conditions are missing.

The fields of impedimetric and potentiometric sensors integrating nanoMIPs for sensing large molecules currently present a wealth of opportunities to explore from several points of view, such as device setup and operation, MIP NPs synthesis procedures and strategies for their immobilization, type of biomolecule to detect, among others.

### Other Methods for Protein Detection

Thermal detection methods recently emerged in the literature as a reliable alternative to optical and electrochemical sensors due to their simple and low cost operation, fast measurement time, and possibility of real-time monitoring.

**Figure 12:**
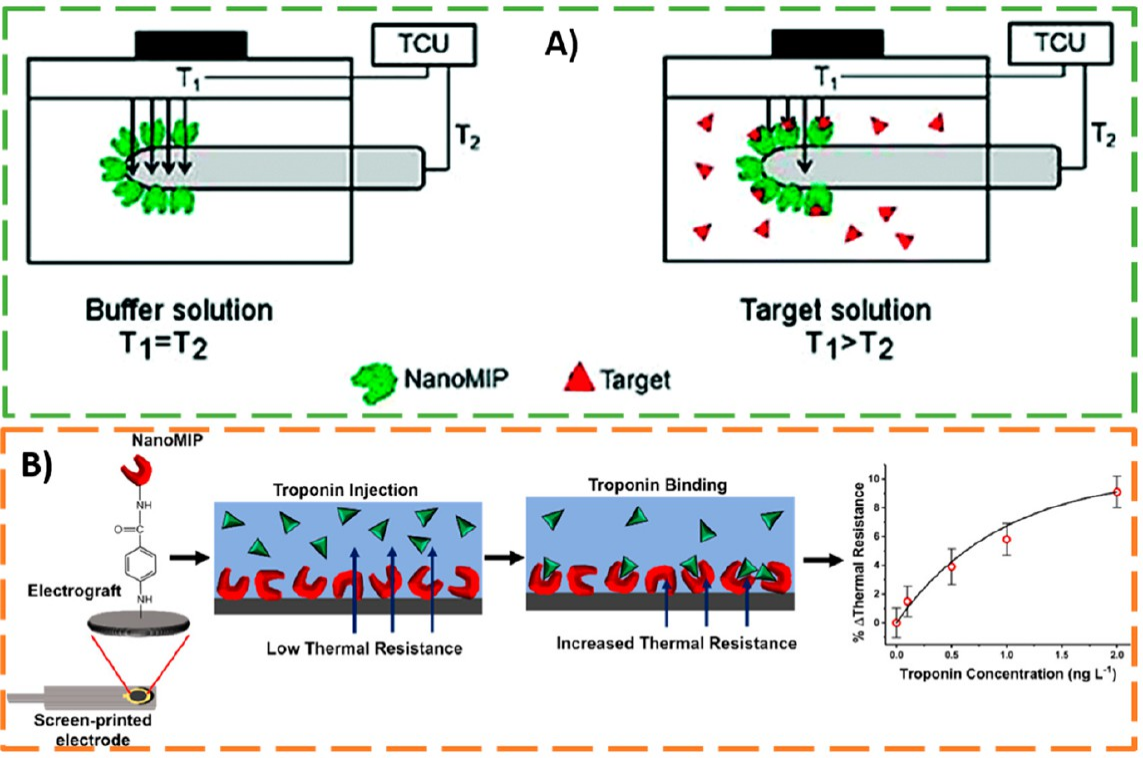
(A) Schematic representation of the thermocouple functionalized with synthetic receptors (T2) by means of dip-coating. TCU represents the temperature control unit of the heat sink (T1; kept constant at 37.00 ± 0.02 °C) and the temperature monitored in the flow cell (T2). When buffer solution circulates in the flow cell, the heat can freely pass through the thermocouple (left), whereas the binding of analyte to MIP NPs blocks the heat-flow in the flow cell, decreasing the measured temperature (right). Adapted with permission from ref (44). Copyright 2018 Royal Society of Chemistry. (B) Schematic representation of the covalent attachment of the nanoMIPs to the SPE surface via electrografting of 4-aminobenzoic acid (4-ABA) followed by EDC/NHS activation of carboxylate groups for amine coupling via reactive esters. Operating principle, based on the increase of thermal resistance after target biomarker binding to the synthetic receptors, and the obtained dose–response curve for troponin I are also shown in the figure. Adapted from ref (24). Copyright 2021 American Chemical Society.

In 2018, Canfarotta et al.^44^ reported for the first time the use of nanoMIPs combined with thermal detection for the bioanalysis of peptides and proteins. The device setup was very simple since it only required a heat-source and two thermocouples (see Figure 12A). One of these thermocouples was previously functionalized with MIP NPs by means of dip-coating and measures the temperature in the flow cell when the synthetic receptors are exposed to running buffer and analyte solutions. The binding of target biomolecule to the MIP receptor layer at the thermocouple inhibits the heat-flow from the sensor to the aqueous phase, thereby decreasing the measured temperature. The overall detection scheme was demonstrated for several analytes with different sizes (biotin, vancomycin, an epitope of epidermal growth factor receptor, and trypsin). The prepared thermal platforms allowed the quantification of target (bio)molecules at physiologically relevant levels (LODs of ∼3–5 nM). With regard to selectivity, a higher thermal response was obtained for target analytes relative to structural analogues or coexisting interferents in the sample matrix (selectivity factors within 1.7–4.5). To test the applicability in the clinical diagnosis context, the MIP-based thermal sensor was successfully applied for epidermal growth factor receptor (EGFR) detection in saliva samples (1:1 diluted in PBS).

A crucial advance in the biomedical field is to move from single to multiplex detection of biomarkers, significantly increasing diagnostic precision and trueness.^119^ To further demonstrate the great potential of thermal detection in the clinical setting, the same research group developed a multiplex device for simultaneous thermal analysis of two cardiac biomarkers, the heart-fatty acid binding protein (H-FABP) and the ST2 (suppression of tumorigenicity 2).^120^ MIP NPs specific for each biomarker were prepared and then dip-cotated onto the surface of thermocouples. Flow cells of different multiplex design incorporating the functionalized thermocouples and an internal control were developed. After optimization, the multiplex sensing platform allowed the detection of both biomarkers at physiologically relevant levels (LODs of few ng mL^–1^). Ultimately, the thermal device was successfully applied for simultaneous quantification of H-FABP and ST2 in spiked fetal bovine serum (FBS) samples, highlighting the thermal analysis as a promising multimarker detection approach for precision medicine.

After these pioneer studies, researchers rapidly found new routes for improvement of this emerging technology by increasing its commercial potential and finding new clinical applications. In this context, McClements et al.^24^ recently introduced innovative substrates for thermal analysis, the screen-printed electrodes (SPEs), and new strategies for effective nanoMIPs immobilization on sensing platforms. The dip-coating method, although experimentally simple, produces irregular MIP layers and suffers from lack of reproducibility. Thus, the authors provided a systematic study on how the immobilization of artificial receptors influences the performance of the thermal assay and explored other strategies for surface functionalization, such as the drop casting and the covalent attachment of the nanoMIPs onto the chip surface. They concluded that the covalent attachment of MIP NPs to the surface of SPEs enhanced the thermal assay reproducibility while benefiting from sensor mass production for widespread applications, including the POC disease diagnosis.^24^ The optimized thermal detection devices were used for detection of the biomarker cardiac troponin I (cTnI). NanoMIPs for specific recognition of cTnI were covalently attached to a graphite SPE through electrochemical grafting of a diazonium salt and EDC/NHS coupling reaction (see Figure 12B). Then, the sensing platforms were exposed to buffered solutions spiked with increasing amounts of cTnI (0.1–2.0 ng L^–1^). The binding of the target biomarker to synthetic receptors increased the measured thermal resistance allowing one to build the dose–response curve for troponin I (see Figure 12B). The obtained LOD (0.46 ± 0.07 ng L^–1^) was inferior to most of the methods reported for cTnI detection. In addition, the prepared nanoMIPs showed good selectivity, being able to distinguish cTnI from BSA, glucose, and even its structural analogue cardiac troponin T (cTnT). Still, studies focusing on biomarker detection in complex biofluids, such as serum, are missing in their work.

Meanwhile, and to face the world pandemics, the optimized thermal detection assay was used for fast, robust, and sensitive screening of SARS-CoV-2 in POC.^25^ In order to reduce reagent costs, a small SARS-CoV-2 fragment (∼10 amino acids) was used as the epitope. The epitope was selected by *in silico* analysis, meaning that, if needed, a new epitope can be easily identified for a new virus variant and the corresponding MIP NPs prepared in a short period. Interestingly, the prepared MIP receptors showed sensing capabilities similar to biological antibodies for the alpha variant, although much less strict storage conditions were needed for the plastic antibodies. Furthermore, the response of nanoMIPs to coexisting interferents in biofluids (open reading frame 8, interleukin-6, and human serum albumin) was significantly reduced when compared to the target virus spike protein. In addition, the thermal assay displayed high detection sensitivity with LODs (of ∼9.9 fg mL^–1^ and ∼6.1 fg mL^–1^ for alpha and delta variants, respectively) remarkably lower than other methods for SARS-CoV-2 detection. Clinical samples consisting of nose and throat swabs from patients symptomatic for COVID-19 were successfully analyzed with the overall signal variation being much larger for COVID-positive patient samples. The portable thermal device was miniaturized to reduce sample volume (to only 100 μL), and samples were analyzed with minimal sample pretreatment. The total assay time was only ∼15 min.

Nanosized hydrogel MIP particles were also employed for detection of another type of pathogenic organisms. Recently, Kushwaha et al.^50^ developed an electrochemical quartz crystal microbalance (EQCM) sensor integrating nanoMIPs for sensing of *Mycobacterium leprae* bacteria through its protein’s epitope (linear amino acid sequence LP-15).

**Figure 13:**
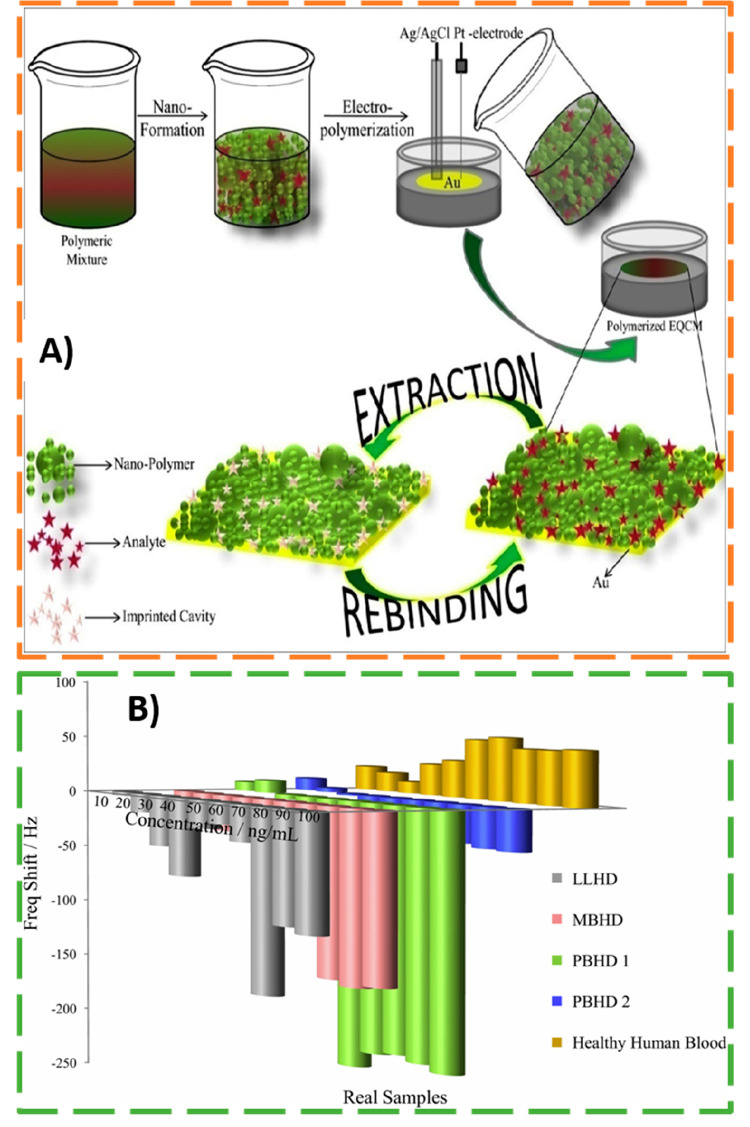
(A) Schematic representation of the MIP-EQCM sensor fabrication process for detection of *Mycobacterium leprae*, consisting of MIP NPs polymerization using multiple functional monomers, their incorporation on the piezoelectric transducer by electropolymerization of conducting poly(4-aminothiophenol), followed by template extraction from the polymeric matrix for formation of binding cavities. (B) EQCM sensor response to real blood samples from healthy human donors and from patients affected by Hansen′s diseases (leprosy), like multibacillary Hansen’s disease (MBHD), pauci-bacillary Hansen′s disease (PBHD), and lepromatous leprosy Hansen′s disease (LLHD). Adapted with permission from ref (50). Copyright 2019 Elsevier B.V.

For preparation of the sensing platforms, nanoMIPs were first prepared by radical polymerization and then incorporated on the gold-coated quartz crystal electrode by electrochemical polymerization of 4-aminothiophenol (4-ATP) added to the prepolymerization mixture (see Figure 13A). The template was removed from the MIP matrix by repetitive exposition of the EQCM electrode to PBS. The extraction procedure was monitored by QCM measurements and confirmed by fluorescence assay. Then, electrochemical and QCM data collected during rebind studies revealed the specific recognition of target analyte (LP-15 epitope) by the imprinted sensor. An imprinting factor (IF) of 8.28 was estimated for an analyte concentration of 140 nM. Moreover, a linear relationship between the frequency shift and the analyte concentration in the range from 10 to 140 nM with a LOD of 0.161 nM was obtained.

In this work, selectivity studies were very well designed by comparing the sensor response for target epitope relative to (i) mismatched amino acids sequences (LP-13, KC-14, VC-13, KW-12, and GW-10) and (ii) proteins present in blood plasma (globulin and albumin). The MIP sensor showed a very good selectivity for the target analyte sequence (LP-15) since no significant response was observed for selected interferents. For example, the sensor response to the target LP-15 sequence was more than four times superior to the response to the LP-13 sequence (with only 2 amino acids less than LP-15). For validation, the piezoelectrogravimmetric sensor was tested against real blood samples from *Mycobacterium leprae* infected patients (see Figure 13B). It is important to notice that the MIP-based EQCM sensor provided a reliable response in real (diluted) complex samples, thus being suitable for the early disease diagnosis of leprosy bacterial infection in the population. Furthermore, the MIP sensor demonstrated high stability over time, allowing multiple cycles of binding-regeneration even 30 days after fabrication, without affecting sensor sensitivity and selectivity.

## Conclusions and Future Perspectives

The advances in nanotechnology opened new perspectives for applications of MIP (nano)materials in chemical sensing. This review focused on an important class of analyte-responsive materials, the hydrogels, which are very appealing for preparation of innovative imprinted (nano)materials for sensing macromolecules, such as peptides and proteins. MIP hydrogel nanospheres can be easily produced by chemical synthesis. To face some challenges due to the large size and complexity of protein templates, MIP recognition mediated by epitopes is currently a reliable strategy to minimize the limitations of whole-protein imprinting.

Several assay formats incorporating nanoMIPs showed high potential for transition from research detection prototypes to viable commercial products. That is the case of MINA that takes advantage of the high stability, cost-effectiveness, easy handling, and storage of imprinted nanomaterials to become a reliable alternative to commercial ELISA kits. Besides, the technological advances introduced in standard ELISA assays with the use of magnetic materials, combined with fluorescence readout, allowed one to achieve ultrasensitive detection levels. Moreover, other opportunities for MIP companies can arise from (bio)applications in the fields of biomedical diagnosis and food control with the production and commercialization of (i) nanoMIPs for integration into plasmonic and thermal sensors for simple, sensitive, and reproducible analysis of proteins in real-time or (ii) fluorescence and SERS-active nanoMIPs for bioimaging of cells and organelles. In addition, other optical sensing technologies compatible with MI technology, such as the lateral flow immunoassays (LFIAs),^121^ remains relatively unexplored. Thus, imprinted nanogels can be coupled to AuNPs or immobilized over the test line of strip sensors for rapid disease diagnosis, performed at home and analyzed by the naked eye.

Electrochemical devices based on MIP NPs currently have the potential to generate a huge impact in the sensor market. In particular, the introduction of self-reporting electrochemical nanoMIPs (e-nanoMIPs), brought in innovative features to amperometric sensors, enabling straightforward and label-free protein bioanalysis. Furthermore, the type, dimension, and design of detection substrates can have a tremendous impact in the overall sensor applicability. A current trend in the biosensing field is the development of portable sensors (using screen-printed electrodes, for example) integrating nanoMIPs for POC disease diagnosis by employing not only electrochemical techniques but also thermal detection, accelerating the developments of this emergent technology with high commercialization potential.

A critical issue in (bio)sensors development is its applicability in real scenario conditions (human biofluids, food matrices, etc.) which can be rather complicated due to nonspecific binding (NSB). To address this issue, nanoMIPs composition can be optimized to enhance the affinity for target biomolecule against coexisting interferents in complex samples. Other strategies that remain unexplored, such as the use of novel monomers having antifouling properties (zwitterionic 2-methacryloyloxyethyl phosphorylcholine,^122^ poly(ethylene glycol) methyl ether methacrylate,^123^ etc.) and new polymerization systems, can potentially reduce NSB to nanoMIPs. Besides, the careful optimization of experimental conditions and well-designed selectivity tests are crucial to improve the sensor performance in real samples. Some inspiring works managed to solve these issues, reporting the detection of peptides, proteins, and pathogens in biological fluids (urine, plasma, serum, and blood) without the need of extensive sample treatment or preconcentration steps. Still, more applied work addressing sensors operating directly in complex samples is needed to encourage more investments on nanoMIPs for widespread biosensing applications. If necessary, an appealing strategy to further improve the analytical performance of MIPs in harsh environments is to combine them with aptamers to form aptaMIPs.^124^ These cross-linked hybrid materials already showed higher binding affinity toward target proteins in comparison to traditional MIP NPs.^125^

Another demand of the (bio)sensing field is to expand to the simultaneous detection of several analytes in the same run aiming to increase diagnostic precision and accuracy. This technological advance was recently introduced with the development of a thermal device enabling the simultaneous analysis of two biomarkers in a single test.^120^ Although MIP hydrogel nanospheres specific for each biomarker were independently prepared to perform the simultaneous recognition, the production of binding cavities of multiple templates in a single MIP NP (multiple-template imprinting)^126^ can be a promising approach for multiplex protein detection in the near future. Alternatively, MIP-aptamer dual recognition systems offers flexibility of design for multianalyte detection by incorporation of chosen aptamer sequences into the MIP structure.

This review aims to demonstrate that hydrogel MIP NPs can have a tremendous impact in the Chemical Sensing field, particularly in the clinical setting with the development of new mobile technologies for POC early disease diagnosis or incorporated into wearable devices for real-time health monitoring, opening the route for a new generation of self-testing healthcare devices.

## List of Acronyms

4-ABA: 4-aminobenzoic acid
4-ATP: 4-aminothiophenol
APS: ammonium persulfate
APTES: (3-aminopropyl)triethoxysilane
APTMS: 3-aminopropyltrimethyloxysilane
B2M: β2 microglobulin
BaP: benzo[a]pyrene
BLG: β-lactoglobulin
BSA: bovine serum albumin
C4-HSL: C4-homoserine lactone
CDs: carbon dots
CIP: cleaning in place
cTnI: cardiac troponin I
cTnT: cardiac troponin T
CV: cyclic voltammetry
Cyt c: cytochrome c
DEA: des-ethyl atrazine
DEDIA: des-ethyl-des-isopropyl atrazine
DIA: des-isopropyl atrazine
DMSO: dimethyl sulfoxide
DPV: differential pulse voltammetry
EDC: 1-ethyl-3-(3-(dimethylamino)propyl)carbodiimide
EGFR: epidermal growth factor receptor
ELISA: enzyme-linked immunosorbent assay
e-MIP: electrochemical MIP
EQCM: electrochemical QCM
FBS: fetal bovine serum
FcMMA: ferrocenylmethyl methacrylate
FITC: fluorescein isothiocyanate
GA: glutaraldehyde
H-FABP: hear-fatty acid binding protein
HRP: horseradish peroxidase
HSA: human serum albumin
HTR: human serum transferrin
IF: imprinting factor
*K*_D_: dissociation constant
LCST: lower critical solution temperature
LDF: light-diffusing fiber
LLHD: lepromatous leprosy Hansen’s disease
LOD: limit of detection
LPG: long period grating
LTE: leukotriene
MALDI-TOF-MS: matrix-assisted laser desorption ionization time-of-flight mass spectrometry
MBHD: multibacillary Hansen’s disease
Mel: melittin
MI: molecular imprinting
MINA: molecularly imprinted polymer nanoparticle-based assay
MIP NPs, nanoMIPs: molecularly imprinted polymer nanoparticles
MIP: molecularly imprinted polymer
MS: mass spectrometry
NHS: *N*-hydroxysuccinimide
NIP: nonimprinted polymer
NIPAAm: *N*-isopropylacrylamide
NPs: nanoparticles
NSB: nonspecific binding
PAB: *p*-aminobenzamidine
PAH: polycyclic aromatic hydrocarbon
PBHD: pauci-bacillary Hansen’s disease
p*I*: isoelectric point
PNIPAAm: poly(*N*-isopropylacrylamide)
POC: point-of-care
POF: plastic optical fiber
PRO: propazine
QCM: quartz crystal microbalance
QDs: quantum dots
RAFT: reversible addition–fragmentation chain transfer
RBD: receptor binding domain
RI: refractive index
SAM: self-assembled monolayer
SDS: sodium dodecyl sulfate
SEM: scanning electron microscopy
SERS: surface-enhanced Raman spectroscopy
SIM: simazine
SPE: screen-printed electrode
SPR: surface plasmon resonance
ST2: suppression of tumorigenicity 2
SWV: square-wave voltammetry
TEMED: *N*,*N*,*N*′,*N*′-tetramethylethylenediamine
THC: tetrahydrocannabinol
TMB: tetramethylbenzidine
VEGF: vascular endothelial growth factor
VFc: vinylferrocene

## Vocabulary

molecular imprinting: technique used to create artificial recognition sites in a polymer matrix with high specificity for a particular analyte by polymerizing functional monomers around a template (bio)molecule
plastic antibodies: synthetic biomimetic materials having molecular recognition abilities for a given analyte similar to natural receptors
hydrogels: three-dimensional, cross-linked networks of hydrophilic polymers that can absorb and retain large amounts of water, exhibiting significant swelling behavior in response to external stimulus
nanoMIPs: common designation for a class of nanoscale imprinted materials, the molecularly imprinted polymer nanoparticles (MIP NPs)
biosensor: self-contained receptor-transducer device that integrates a biological receptor to provide selective and quantitative or semiquantitative analytical information, usually an analyte concentration
